# The Influence of Charge Correlation and Ion Solvation on the Phase Behavior of Single-Ion Conducting Polymer Blend Electrolytes Using SAXS/SANS

**DOI:** 10.1021/acs.macromol.5c00860

**Published:** 2025-08-11

**Authors:** Hsin-Ju Wu, Lilin He, William M. Breining, David M. Lynn, Whitney S. Loo

**Affiliations:** † Department of Chemical and Biological Engineering, 278762 University of Wisconsin—Madison, 1415 Engineering Drive, Madison, Wisconsin 53706, United States; ‡ Neutron Scattering Division, 6146 Oak Ridge National Laboratory, Oak Ridge, Tennessee 37831-6393, United States; § Department of Chemistry, University of Wisconsin—Madison, 1101 University Avenue, Madison, Wisconsin 53706, United States

## Abstract

Single-ion conducting polymer blends (SICPBs) have demonstrated exceptional electrochemical performance as solid-state battery electrolytes; however, their nanoscale morphology and thermodynamic behavior remain unexplored. In this work, we investigate blends composed of deuterated poly­(ethylene oxide) and poly­[lithium sulfonyl­(trifluoromethane sulfonyl)­imide methacrylate], dPEO/P­(LiMTFSI), and report the first experimental study of the nanostructures of charge-neutral polymer blends using small-angle neutron scattering (SANS) and small-angle X-ray scattering (SAXS). Despite the macroscopic miscibility indicated by a single glass-transition temperature, SANS and SAXS results reveal disordered, charge-correlated nanostructures that are strongly influenced by blend composition and temperature. At low concentrations of charge polymer, the scattering is dominated by concentration fluctuations, and the random phase approximation is applied to extract values of the Flory–Huggins interaction parameter, χ_SC_. At higher charged polymer content, concentration fluctuations are suppressed, and a correlation model is used to characterize the nanostructures of the charge correlations. We find that the structures of the charge correlations are highly dependent on blend composition—consistent with predictions from Sing’s self-consistent field theory-liquid state models. Understanding these features is essential for uncovering the ion transport mechanism that leads to improved electrochemical performance previously reported in SICPB systems.

## Graphical abstract

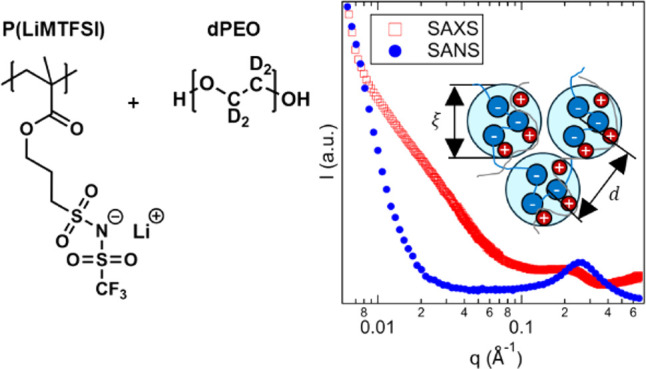

## Introduction

Polymer electrolytes have emerged as promising solid-state materials for energy storage due to their flexibility and safety advantages.^1^ Poly­(ethylene oxide) (PEO)/lithium bis­(trifluoromethanesulfonyl) imide (LiTFSI) systems have been widely studied for solid-state battery applications due to their high ionic conductivity.^2,3^ However, PEO-based electrolytes typically suffer from low cation transference numbers with recent reports demonstrating negative cation transference numbers at high salt concentrations (*r* = [Li^+^]/[EO] ≥ 0.12).^4,5^ This phenomenon was attributed to the formation of concentrated ion clusters that ultimately hinder battery performance.^6^ One strategy to improve the cation transference number is to eliminate ion concentration gradients by reducing the mobility of the negatively charged ions.^7^ Single-ion conducting polymers (SICPs), wherein anions are immobilized via covalent bonds to the polymer backbone, mitigate the formation of concentration gradients and promote lithium-ion motion.^8^ Nevertheless, SICPs often exhibit low ionic conductivities since the high glass-transition temperatures, *T*_g_, typical of SICPs restrict segmental motion and further hinder ion transport.^8^ To overcome the trade-off between ionic conductivity and cation transference numbers, researchers have developed single-ion conducting polymer blends (SICPBs), which consist of an ion-containing polymer and an ion-conducting polymer. Previous studies on SICPBs have highlighted enhanced electrochemical performance including superionic transport^9^ as well as improved electrochemical stability.^10^ Although nanostructure is known to strongly influence ion transport,^11,12^ these studies on SICPBs have assumed that miscible blends, identified by a single *T*_g_, form a homogeneous phase rather than directly investigating their nanoscopic morphology.

The combination of small-angle neutron scattering (SANS) and small-angle X-ray scattering (SAXS) can provide complementary information on nanoscale morphology.^13–16^ Specifically, neutrons provide good contrast between elemental isotopes, such as hydrogen and deuterium, making SANS particularly useful for studying hydrogen-containing materials like macromolecules. In contrast, X-rays are sensitive to electron density and are more suitable to distinguish between light and heavy elements. SANS results can be fitted to theoretical models in order to experimentally measure the Flory–Huggins interaction parameter, *χ*, between polymer chains and elucidate phase behavior at nanometer length scales.^13,14,17–21^ Although no experimental SANS data currently exist for SICPB systems, several theories have been developed to describe the thermodynamics and nanostructures of polymer blends including salt-doped neutral homopolymer blends and polyelectrolyte blends.^22–27^ One theory developed by Fredrickson and coworkers described the phase behavior of polyelectrolyte blends comprised of two oppositely charged polyelectrolytes.^25,28^ Through the construction of a field-theoretic model that accounts for dielectric contrast and ion solvation effect, they showed that the competition between electrostatics and counterion entropy can lead to either macro- or microphase separation. The oppositely charged polyelectrolytes form electrostatically stabilized microphases with morphologies similar to those observed in block copolymers, e.g., lamellar, body center cubic, and hexagonally packed cylinder phases. Another theory developed by Sing and Olvera de la Cruz^22,29,30^ incorporates charge correlation from liquid state theory (SCFT-LS) within the random phase approximation (PRA) framework previously developed by de Gennes^31,32^ to derive a new expression for the structure factor of SICPBs, i.e., charge-neutral polymer blends. This expression extends RPA for uncharged binary polymer blends by introducing correction terms, characterized by a new parameter, α, to account for electrostatics through an effective interaction parameter, χ_eff_ = χ – α, which reflects the local charge structure.^30^ The results of that past study show that χ_eff_, and therefore α, is highly dependent on polymer blend properties, including blend composition, electrostatic interaction strength, and the charge density of the charged polymer component.

This study aimed to experimentally probe the phase behavior of SICPBs and compare our findings with previously developed theoretical models. We prepared blends of two polymers, deuterated PEO (dPEO) and poly­[lithium sulfonyl­(trifluoromethane sulfonyl)­imide methacrylate] (PLiMTFSI), to investigate the effect of mixing ratio, *r*, temperature, and dPEO molecular weight on the nanostructure of SICPBs using SANS and SAXS. All blends prepared for this study were found to be macroscopically miscible, with each exhibiting a single *T*_g_. The SANS profiles were dominated by contrast between the hydrogenated backbone of P­(LiMTFSI) and the deuterated backbone of dPEO, highlighting interactions between polymer chains, while SAXS profiles primarily capture scattering from the fluorine-containing anions. By comparing results from both techniques with a theoretical model inspired by the work of Sing and coworkers,^29,30^ we find that the interplay between ion solvation and electrostatic interactions governs the nanostructure and phase behavior of the blends. For instance, blends with low ion concentrations exhibited lower critical solution temperature (LCST) behavior, while blends with higher ion concentrations displayed upper critical solution temperature (UCST) behavior. Through this study, we provide the first experimental dataset that investigates the nanostructures of charge-neutral polymer blends and demonstrate that the blend phase behavior is highly dependent on blend composition.

## Materials and Methods

### Chemicals

Potassium 3-sulfopropyl methacrylate (98%, Sigma-Aldrich, USA), oxalyl chloride (98%, Sigma-Aldrich, USA), potassium bifluoride (KHF_2_, 99%, Sigma-Aldrich, USA), trifluoromethanesulfonamide (95%, Sigma-Aldrich, USA), acetonitrile (ACN, ≥99.5%, Sigma-Aldrich, USA), dimethylformamide (DMF, anhydrous, 99.8%, Sigma-Aldrich, USA), dichloromethane (DCM, anhydrous, ≥99.8%, contains 40–150 ppm amylene as stabilizer, Sigma-Aldrich, USA), diethyl ether (Fisher Scientific, USA), 2-cyano-2-propyl benzodithioate (>97%, Sigma-Aldrich, USA), acetone (≥99.5%, Sigma-Aldrich, USA), dimethyl sulfoxide-*d*_6_ (DMSO-*d*_6_, 99.9 atom % D, contains 0.03% (v/v), Sigma-Aldrich, USA), sodium sulfate (Na_2_SO_4_, ≥99.0%, anhydrous, Sigma-Aldrich, USA), calcium carbonate (CaCO_3_, ≥99.0%, Sigma-Aldrich, USA), butylated hydroxytoluene (BHT, ≥99%, Sigma-Aldrich, USA), potassium carbonate (K_2_CO_3_, ≥99.0%, Sigma-Aldrich, USA), calcium gluconate (monohydrate, DOT Scientific, USA), methanol (≥99.8%, Sigma-Aldrich, USA), lithium chloride (LiCl, 99%, Sigma-Aldrich, USA), and sodium chloride (NaCl, ≥99%, Sigma-Aldrich, USA) were used as received. Azobis­(isobutyronitrile) (AIBN, Sigma-Aldrich, USA) was purified by recrystallization from methanol, involving dissolution at 50 °C followed by cooling in a freezer to induce crystal formation. Deuterated poly­(ethylene oxide) (dPEO, Polymer Source, Canada) was dried under vacuum in the antechamber of a glovebox for 24 h and then transferred into the glovebox.

### Monomer Synthesis

The three-step synthesis of the potassium sulfonyl­(trifluoromethane sulfonyl)­imide methacrylate (KMTFSI) monomer was performed following the procedure reported by Lee^33^ as outlined in Scheme S1. In the first step, 120 mL of anhydrous DCM was added to a dried 250 mL three-neck Schlenk flask under a nitrogen atmosphere. The solution was cooled to 0 °C, and a catalytic amount of DMF (2 mL) was added dropwise, followed by the slow addition of oxalyl chloride (9.5 mL), during which gas release was observed. After stirring for 30 min at room temperature, the mixture was cooled to 0 °C and 20 g of potassium 3-sulfopropyl methacrylate and 10 mg of BHT were added under a positive nitrogen atmosphere. The reaction proceeded overnight at room temperature in the dark, then quenched with water. The organic layer was extracted and washed thoroughly with deionized water five times, followed by two washes with saturated NaCl solution (brine). The organic layer was dried over anhydrous Na_2_SO_4_ until free-flowing crystals formed, then filtered and concentrated by rotary evaporation. The resulting transparent dark yellow oil of 3-propylsulfonyl chloride methacrylate was obtained (18.27 g, 99.3% yield).

In the second step, 3-propylsulfonyl chloride methacrylate was dissolved in 80 mL of ACN in a 250 mL Nalgene beaker. Separately, 12.6 g of KHF_2_ was dissolved in 35 mL of deionized water in a 250 mL of perfluoroalkoxy alkane (PFA) round-bottom flask. The ACN solution was added slowly to the KHF_2_ solution using a polypropylene funnel and the reaction mixture was stirred at room temperature for 4 h. Afterward, the solution was poured into a 1 L Nalgene beaker and diluted with 200 mL of DCM. 140 mL of deionized water was added to promote phase separation. The organic and aqueous layers were separated using a separatory funnel. The aqueous layer was immediately diluted and neutralized with CaCO_3_ in plastic containers. The DCM layer was washed thoroughly with deionized water five times, followed by two washes with brine. It was then dried over anhydrous Na_2_SO_4_, filtered, and concentrated by rotary evaporation to yield 3-propylsulfonylfluoride methacrylate as a clear, light-yellow oil (16.30 g, 96.2% yield).

Finally, 3-propylsulfonylfluoride methacrylate and 9.5 g of trifluoromethanesulfonamide were dissolved in 65 mL of ACN in a 100 mL three-neck glass flask. 29.0 g of K_2_CO_3_ was added, and the mixture was refluxed at 65 °C under nitrogen overnight. After completion, the mixture was filtered to remove K_2_CO_3_ solids, and the filtrate was concentrated via rotary evaporation. The crude product was recrystallized by DCM to yield white KMTFSI powder (10.10 g, 86% yield). ^1^H- and ^19^F-NMR spectra were confirmed in DMSO-*d*_6_ using a Bruker AVANCE III 400 NMR spectrometer as shown in Figures S1 and S2, respectively.

### P­(LiMTFSI) Synthesis

Poly­[potassium sulfonyl­(trifluoromethane sulfonyl)­imide methacrylate], P­(KMTFSI), was synthesized by reversible addition–fragmentation chain-transfer polymerization (RAFT) (Scheme S2). Briefly, KMTFSI, azobis­(isobutyronitrile), and 2-cyano-2-propyl benzodithioate were fully dissolved in DMF (8.5 mL) in a 25 mL Schlenk flask. After three freeze–pump–thaw cycles, the flask was filled with nitrogen and heated at 70 °C for 24 h. The product was precipitated into diethyl ether and recovered using methanol three times. The polymer was dried at 60 °C under a high vacuum for 24 h. Gel permeation chromatography (GPC) experiments were conducted using a Wyatt Dawn Heleos multiangle light scattering (MALLS) and a Wyatt Optilab T-Rex refractive index (RI) detector. Two in-line columns were used: an Agilent PLgel 10 μm 10^3^ Å column and an Agilent Resipore column. The molecular weight and polydispersity index, *Đ*, of P­(KMTFSI) were measured using DMF as the mobile phase with a flow rate of 1 mL/min (Figure S5). The GPC results of homopolymers are summarized in Table S1. ^1^H- and ^19^F-NMR spectra were recorded in dimethyl sulfoxide-*d*_6_ using a Bruker AVANCE III 400 NMR spectrometer (Figures S3 and S4).

P­(KMTFSI) and 4 equiv of 0.2 M LiCl were stirred at room temperature overnight to facilitate ion exchange of potassium ions with lithium ions. The excess lithium chloride was removed by dialysis against deionized (DI) water (MWCO: 1 kDa) for 3 days. The resulting light pink P­(LiMTFSI) powder was obtained after freeze-drying for 4 days and subsequently stored in an argon glovebox (MBraun) with water and oxygen levels maintained at less than 1 ppm.

### Preparation of Polymer Blends

The SICPB system investigated here consists of deuterated PEO (dPEO) and P­(LiMTFSI), where dPEO was utilized to enhance neutron contrast and facilitate structural characterization. A series of dPEO/P­(LiMTFSI) blends with varying mixing ratios, *r* = [P­(LiMTFSI)]/[dPEO], and polymer molecular weights were prepared in a glovebox, with their compositions summarized in Table S2. P­(LiMTFSI) and dPEO were dissolved in anhydrous methanol separately to a concentration of 10 mg/mL and stirred overnight. The solutions were blended and stirred at 70 °C for a minimum of 12 h. Once the solutions were fully mixed, the caps were removed from the vials and the solvent was allowed to evaporate. After drying on a hot plate at 70 °C overnight, the polymer blends were transferred into a vacuum oven and dried under vacuum for 72 h at 90 °C to remove the residual solvent.

### Differential Scanning Calorimetry (DSC) Analysis

Polymer samples ranging from 2 to 10 mg were hermetically sealed in aluminum pans in a glovebox. DSC experiments were performed using a TA Instruments Q100 instrument using two heating and cooling cycles. The heating rate was 10 °C/min and the cooling rate was 5 °C/min over a temperature range of −80 to 200 °C. The melting temperature (*T*_m_) and glass transition temperature (*T*_g_) were determined from the second heating cycle.

### Small-Angle Neutron Scattering (SANS) Measurements

The dPEO/P­(LiMTFSI) blends were prepared by melting into a copper 3.5 cm × 4.5 cm × 2.2 cm sample holder in a glovebox and then sandwiched between two 2.54 cm outer diameter quartz windows with 1.66 mm spacing. The assembled samples were degassed in the glovebox antechamber at 110 °C for 30 min. Temperature-dependent SANS experiments were conducted on the GP-SANS (CG-2) beamline at Oak Ridge National Lab (ORNL), Neutron Science Directorate.^34^ The SANS experiments were performed at 70, 90, 110, and 130 °C with a *q* range of 0.005 to 0.5 Å^–1^. The SANS intensity, *I*(*q*), was recorded as a function of the magnitude of the scattering wavevector, defined as $q=\frac{4\pi }{\lambda }\sin\left(\frac{\theta }{2}\right)$ , where *θ* is the scattering angle and λ is the wavelength of the neutron beam. Background scattering from the instrument and sample holder was subtracted during data reduction and standard corrections were applied for detector efficiency, sample thickness, and transmission. The final scattering intensity was placed on an absolute scale using a precalibrated standard.^35^

### Small-Angle X-ray Scattering (SAXS) Measurements

The dPEO/P­(LiMTFSI) blends were prepared by melting the polymer blends at 90 °C into a stainless-steel holder with a 4 mm inner diameter and a 0.554 mm wall thickness. The samples were annealed at 90 °C in a vacuum oven for at least 24 h to remove bubbles. After this, the heater was turned off and the samples were slowly cooled under vacuum. Kapton polyimide tape was used to seal the prepared samples. Temperature-dependent SAXS measurements were performed using a Xeuss 3.0 system. Measurements were performed every 20 °C from 50 to 130 °C with an exposure time of 900 seconds in high-intensity mode. Samples were allowed to equilibrate at each temperature for 20 minutes prior to measurement. The resulting 2D scattering patterns were isotropic and were azimuthally integrated into 1D profiles using XSACT Pro advanced data analysis software. Background intensities from the Kapton tape were subtracted from the total intensity. The SAXS intensity, *I*(*q*) , was recorded as a function of the magnitude of the scattering wavevector, defined as $q=\frac{4\pi }{\lambda }\sin\left(\frac{\theta }{2}\right)$ , where *θ* is the scattering angle and λ is the X-ray wavelength.

## Results and Discussion

### Blend Preparation and Characterization

The SICPB system investigated here consists of deuterated PEO (dPEO) and poly­[lithium 3-(methylacryloxy)­propylsulfonyl-1-(trifluoromethanesulfonylimide)] (P­(LiMTFSI)); the structures of these polymers are shown in Figure 1. The poly­(methyl methacrylate) (PMMA)-based single-ion conducting polymer was chosen to increase miscibility as blends of PEO and PMMA are known to have negative values of χ.^18,36^ The molecular weights and polydispersity indices of homopolymers are listed in Table S1, while the polymer blends were prepared as detailed in Table S2. Sample names are defined as “*M*_n,dPEO_/*M*_n,P(LiMTFSI)_/*r*”, where *r* is the mixing ratio is calculated as the molar ratio of lithium ions to deuterated ethylene oxide monomers, given by *r* = [Li^*+*^]/[dEO] = [P­(LiMTFSI)]/[dPEO]

**Figure 1:**
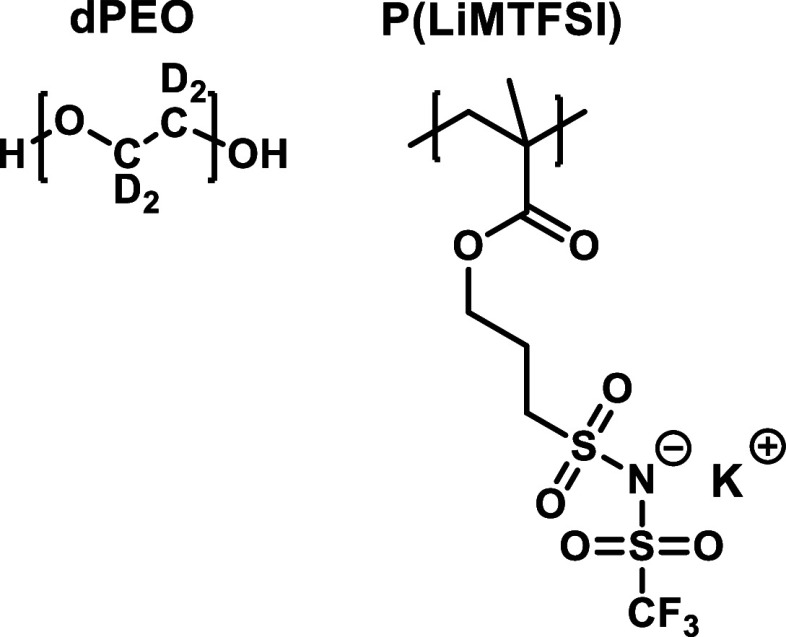
Chemical structures of the deuterated poly­(ethylene oxide) (dPEO) and poly­[lithium sulfonyl­(trifluoromethane sulfonyl)­imide methacrylate] (P­(LiMTFSI)) SICPB system used in this study.

Differential scanning calorimetry (DSC) was used to measure the thermal properties of polymer blends and screen for miscibility. Figure 2 shows the DSC results for blends of 30 kDa dPEO and P­(LiMTFSI). The *T*_g_ of pure P­(LiMTFSI) was measured to be 112.4 °C, while the *T*_g_ of dPEO was measured to be −48.5 °C. Neat dPEO is semicrystalline with a *T*_m_ of 62.5 °C. All blends exhibited a single *T*_g,blend_ between two homopolymers, which can be attributed to the attractive ion–dipole interactions between P­(LiMTFSI) and dPEO.^9^ The *T*_g,blend_ increases with increasing values of *r* , i.e., increasing concentration of P­(LiMTFSI), consistent with the findings of Olmedo-Martínez et al.,^37^ with the presence of a single *T*_g,blend_ being widely accepted as evidence for macroscopic miscibility, indicating that the system is not macrophase separated, i.e., forms a single-phase.^38,39^ Similar to PEO/LiTFSI blends,^40^ the crystallinity of dPEO is suppressed with increasing P(LiMTFSI) content. For *r* > 0.10, no *T*_m_ was observed, indicating that the blends are fully amorphous. A sharp *T*_m_ peak was observed when *r* = 0.05, suggesting a coexistence of dPEO-rich semicrystalline domains and an amorphous phase entangled with P­(LiMTFSI) below *T*_m_. DSC results for 10 kDa dPEO/P­(LiMTFSI) blends shown in Figure S6a followed similar trends. The weak melting peak for *r* = 0.10 indicates minimal crystallinity, consistent with the behavior observed in the 30 kDa blends.

**Figure 2:**
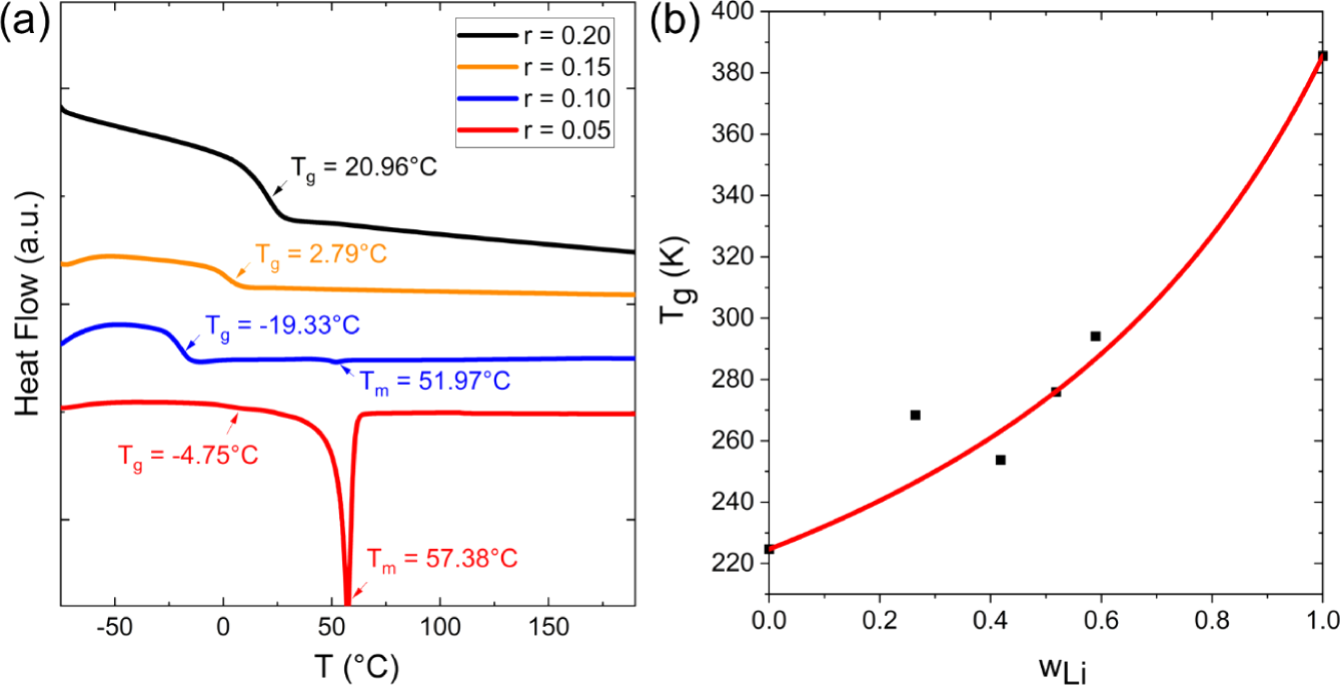
(a) Differential scanning calorimetry (DSC) traces of 30 kDa dPEO/P­(LiMTFSI) blends at various values of *r*. (b) The *T*_g,blend_ of 30 kDa dPEO/P­(LiMTFSI) as a function of weight fraction of P­(LiMTFSI), *w*_Li_. The red solid line represents the fit using the Gordon–Taylor equation.

The effect of blend composition, quantified by the weight fraction of the P­(LiMTFSI), *w*_Li_ , on the thermal transitions described by *T*_g,blend_ is shown in Figure 2b. The black squares represent *T*_g,blend_ values taken from DSC thermograms in Figure 2. The Gordon–Taylor equation has been used previously to describe *T*_g,blend_ in ion-containing polymer blends, as it accounts for nonideal mixing behavior through a fitted parameter, k, which reflects interactions between the polymer components.^9,10^ The solid line in Figure 2b represents the fit using the Gordon–Taylor equation according to eq 1:

1 $$T_{g,\mathrm{blend}}=\frac{w_{1}T_{g,1}+kw_{2}T_{g,2}}{w_{1}+kw_{2}}$$

where *w*_1_ and *w*_2_ are the weight fractions of dPEO and P­(LiMTFSI), *T*_g,1_ and *T*_g,2_ are the *T*_g_ values of dPEO and P­(LiMTFSI), and *k* is an adjustable parameter. We excluded the $T_{g,\mathrm{blend}}$ value at *r* = 0.05 from the fit in Figure 2b, as it is well-known that the presence of semicrystalline domains can affect the observed value for *T*_g_.^41^ The fitted parameter, *k* , quantifies the strength of intermolecular interactions between the polymer components.^42^ For 30 kDa dPEO/P­(LiMTFSI) blends, the value of *k* was determined to be 0.44 ± 0.04, indicating intermediate ion-dipole interactions between dPEO and PLiMTFSI. This value matches previous results reported for similar ion-containing polymer blends.^10,43^ Additional thermal data for blends prepared with 10 kDa dPEO are shown in Figure S6b. Decreasing the molecular weight of dPEO from 30 kDa to 10 kDa shifts the values of *T*_g,blend_ to lower temperatures by approximately 1–2 °C for each blend composition because of the difference in the *T*_g,dPEO_ values with different *M*_n.dPEO_. The fitted *k* value for 10 kDa dPEO/P­(LiMTFSI) blends was 0.50 ± 0.04, which is quantitatively similar to the value for the 30 kDa dPEO/PLiMTFSI blends, indicating that the intermolecular interactions are not significantly affected by dPEO molecular weight. Previous work has demonstrated that the molecular weight of P­(LiMTFSI) significantly influences thermal properties of similar blends.^10^ Therefore, our results suggest that the intermolecular interactions responsible for nonideal mixing originate primarily from the P­(LiMTFSI) moieties. While tuning the dPEO properties can alter the segmental dynamics of the blend, as indicated by changes in *T*_g,blend_, it does not substantially change the degree of nonideal mixing in SICPBs because dPEO acts primarily as a solvent in these systems.

### Resolving Nanoscopic Structure with SANS and SAXS

As part of this study, we aimed to provide experimental data sets that could be used to investigate the nanostructures of charged-neutral polymer blends (i.e., SICPBs) using both SANS and SAXS. The thermodynamic behavior of SICPBs is governed by a combination of polymer backbone interactions, ion solvation, and electrostatic interactions. The SANS scattering contrast arises primarily from differences between the deuterated PEO backbone and the hydrogenated P­(LiMTFSI) backbone and, therefore, enables the determination of: (1) the miscibility of the polymer blends, (2) the presence of charge correlations, and (3) the experimental value of the Flory–Huggins interaction parameter taken from scattering, *χ*_SC_. Figure 3a shows the SANS profiles of 30 kDa dPEO/P­(LiMTFSI) blends at 90 °C for various values of *r*. Inspection of these profiles reveals the qualitative features of these SANS profiles to depend strongly on *r*. For example, the scattering contribution from the polymer chains dominates at *r* = 0.05 (red trace), reflecting disordered concentration fluctuations.^20^ While the observation of a single *T*_g_, as discussed above, suggests that the blends are macroscopically miscible, the SANS profiles indicate a nanoscale disordered structure similar to what is observed in block copolymers at low values of segregation strength, *χN*. We hypothesize that this disordered structure arises from nanoscale phase separation between the semicrystalline and amorphous domains, as suggested by the DSC traces (Figure 2a). Previous experimental studies have also observed nanoscale phase separation between the semicrystalline PEO domains in optical micrographs in different SICPBE systems at high PEO concentrations.^44^ As *r* increases to 0.10 (blue trace) and more P­(LiMTFSI) is introduced to the system, electrostatic interactions become more prominent and a correlation peak emerges in the high-*q* region. The disappearance of the mid-*q* feature in the SANS profile suggests that concentration fluctuations are suppressed due to strong ion-dipole interactions. Similar effects have been observed in lithiated single-ion conducting block copolymers^45^ and polyamide/ionomer blends,^14^ where ion dissociation and strong intermolecular interactions enhance miscibility. With further increasing in P­(LiMTFSI) concentration, i.e., increasing *r* (orange and black traces), the high-*q* correlation peak broadens and shifts to higher *q*-values. These changes suggest that the average distance between charge correlations decreases and the correlations become more polydisperse. Theoretical predictions by Sing and coworkers using a hybrid of self-consistent field theory-liquid-state theories (SCFT-LS) also found that strong local ion correlations at low polyelectrolyte content (analogous to low *r* in our system) in charge-neutral polymer blends can lead to phase separation.^22,29,30^ Across all blend compositions, the low-*q* region of the SANS profiles consistently exhibits a *q* ^–4^ power-law behavior, which was previously observed for other ion-containing polymers and was attributed to voids^13,46^ resulting from insufficient degassing and may not reflect intrinsic structural features of the blends.^18,47,48^ Additional SANS data for blends prepared with 10 kDa dPEO are provided in Figure S8a and show qualitatively similar results.

**Figure 3:**
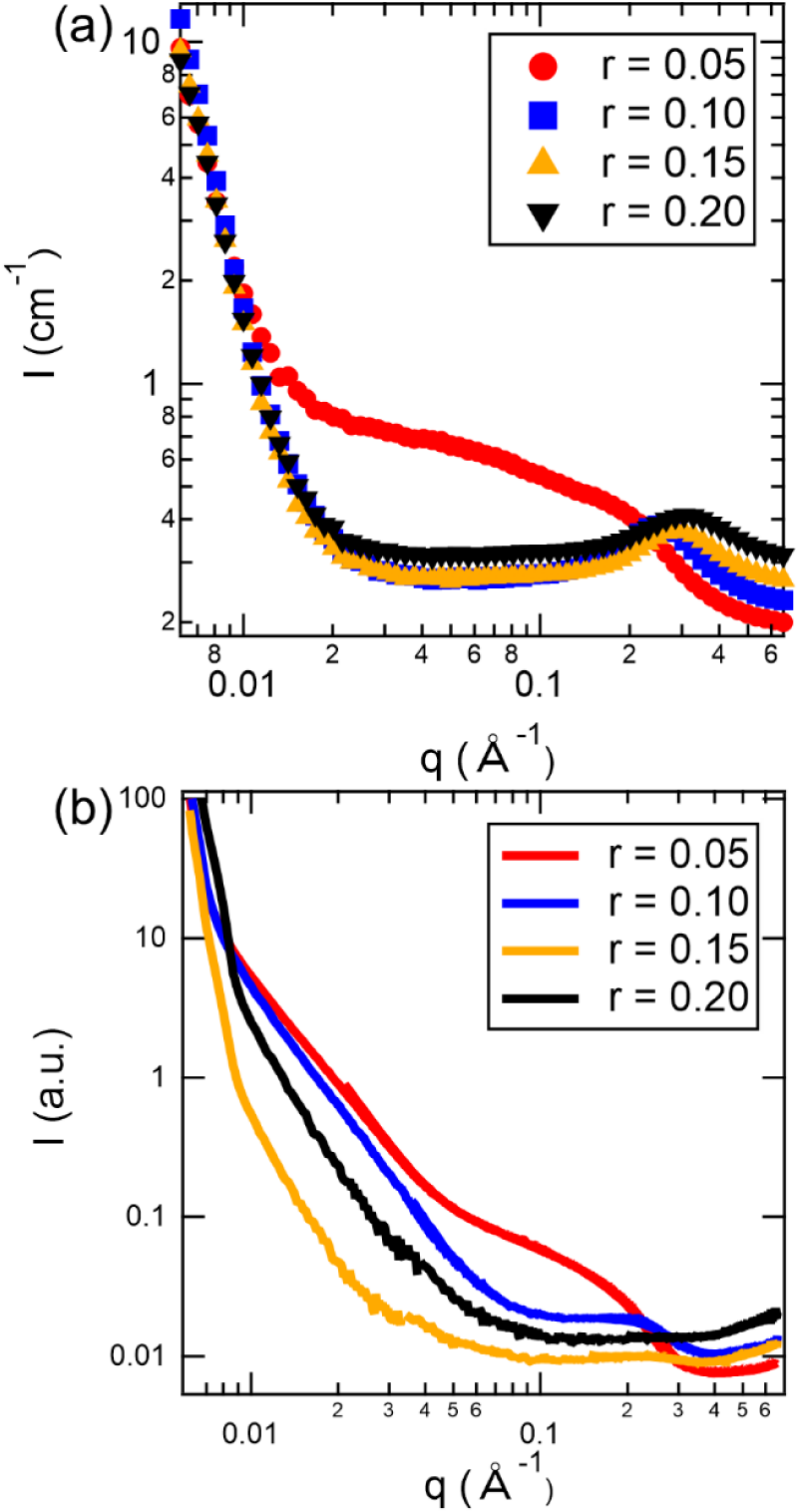
(a) Small-angle neutron scattering (SANS) and (b) small-angle X-ray scattering (SAXS) profiles for 30 kDa dPEO/P­(LiMTFSI) blends at various mixing ratios, *r*, at 90 °C. Error bars in (a) represent the standard deviation and are smaller than the data points.

Figure 3b shows the SAXS profile of 30 kDa dPEO/P­(LiMTFSI) blends at various values of *r* at 90 °C. The SAXS contrast depends on the electron density differences within the system, and thus, provides information regarding the distribution of the tethered anions in the melt phase. At *r* = 0.05 (red trace), an obvious shoulder appears at around *q* = 0.2 Å^–1^, reflecting the presence of ionic domains with an average interdomain spacing of ∼3.1 nm. Similar features have been reported in SAXS profiles for single-ion conducting block copolymers, with an ionomer peak around *q* = 0.25 Å^–1^.^45^ Therefore, the SAXS data reported here are consistent with our DSC and SANS results, suggesting that the formation of semicrystalline domains in the SICPBs can drive nanoscale phase separation. Our results also suggest that when the blend is heated above *T*_m_, the ordered crystalline structure breaks down and attractive ion–dipole interactions between dPEO and lithium-ions promote mixing between the two polymers. As a result, the combination of disordered dPEO/P­(LiMTFSI) entanglements and charge correlations gives rise to nanostructures that are polydisperse in size and shape and heterogeneously distributed throughout the blend. This interpretation is consistent with the broad shoulder observed by SAXS and the disordered concentration fluctuations observed by SANS.

As charge concentration increases to *r* = 0.10 (blue trace) in Figure 3b, the shoulder in mid-*q* region becomes weaker and the low-*q* slope becomes steeper, suggesting a reduction in concentration fluctuations. At *r* ≥ 0.15 (orange and black traces), the SAXS profile becomes featureless without an evident shoulder, implying that the blends become more homogeneously disordered at higher charge concentrations. This apparent increase in miscibility is likely due to the preferential solvation of lithium-ions by the dPEO chains over the P­(LiMTFSI) chains, which has been previously observed in single-ion conducting block copolymers.^49,50^ Segalman and coworkers also observed similar behavior in dry ionic blends of conjugated polyelectrolytes and polymeric ionic liquids,^28^ where increased charge density stabilized the blends and increased miscibility. Based on the SANS and SAXS data, we believe that all SICPB compositions studied form disordered phases that are macroscopically miscible yet exhibit nanoscale heterogeneity. This suggests that our blends lie below the order–disorder transition on the phase diagram, i.e., at low *χN* .^25^ Similar trends are observed for 10 kDa dPEO blends, as shown in Figure S8b. We find that both charge correlations and ion-dipole interactions likely play vital roles in governing the thermodynamics and resulting nanoscale morphology of the SICPBs.^23^

To quantitatively extract structural insights from our SANS data, we developed a composite model inspired by Sing’s theory for charge-neutral polymer blends^30^ as shown by eq 2. This composite model includes: (1) a power-law term for low-*q* region, (2) random phase approximation (RPA) for an uncharged system in the mid-*q* region, and (3) a Lorentzian peak model for high-*q* features induced by charge correlations.

2 $$I_{\mathrm{total}}\left(q\right)=I_{\mathrm{power}}\left(q\right)+I_{\mathrm{RPA}}\left(q\right)+I_{\mathrm{Lorentzian}}\left(q\right)+I_{\mathrm{incoherent}}$$

The contribution from the incoherent scattering, *I*_incoherent_ , was determined using previous methods.^13^ The de Gennes’s RPA theory accounts for the mid-*q* scattering and is commonly used to describe miscible binary polymer blends. It allows for extraction of *χ*_SC_ from scattering data as shown in eq 3:^18,51,52^

3 $$I_{\mathrm{RPA}}\left(q\right)=v_{\mathrm{ref}}{\left(B_{\mathrm{dPEO}}-B_{P\mathrm{LiMTFSI}}\right)}^{2}{[\frac{1}{S_{11}}+\frac{1}{S_{22}}-2\chi_{\mathrm{SC}}]}^{-1}$$

where υ_ref_ is the reference volume (taken here to be 0.1 nm^3^),^17–19^ *B*_*i*_ is the scattering length density of the species *i* , *S*_*ii*_ is the ideal structure factor of species *i* ,^13^ and χ_SC_ is the Flory–Huggins interaction parameter, which quantifies the strength of repulsion between different polymer chains. *B*_*i*_ is given by eq 4:

4 $$B_{i}=\frac{b_{i}}{v_{i}}$$

where *b*_*i*_ is the neutron scattering length and υ_*i*_ $v_{i}$ is the molar monomer volume of species *i*. The neutron scattering lengths for dPEO and P­(LiMTFSI) are 4.58 × 10^–12^ cm and 7.70 × 10^–12^ cm, respectively. The molar monomer volume for dPEO was taken from the literature as 41.34 cm^3^/mol^18^ and the molar volume of P­(LiMTFSI) was calculated using the group contribution approach from van Krevelen et al. to be 211.8 cm^3^/mol.^49–53^ The ideal structure factors, *S_ii_* , are given by eq 5:

5 $$S_{ii}=\varphi_{i}N_{i}P_{i}\left(q\right)$$

where φ_i_ is the volume fraction of species *i* , *N*_i_ is the degree of polymerization of species *i*, and *P*_*i*_(*q*) is the form factor of species *i*. In this study, we choose to use a form factor for a Gaussian coil as given by eq 6:

6 $$P_{i}\left(q\right)=\left[\frac{\exp\left(-q^{2}{R_{g,i}}^{2}\right)-1+q^{2}{R_{g,i}}^{2}}{{\left(q^{2}{R_{g,i}}^{2}\right)}^{2}}\right]$$

where *R*_*g,i*_ is the radius of gyration of species *i* according to eq 7:

7 $${R_{g,i}}^{2}=\frac{\alpha_{i}N_{i}{l_{i}}^{2}}{6}$$

where α_*i*_ is a chain stretching parameter that is used as a fitting parameter and *l*_*i*_ is statistical segment length of species *i*. The value of *l*_dPEO_ is 7.2 Å and the value of *l*_P(LiMTFSI)_ is approximated by the value for PMMA, which is 5.8 Å.^18^

The correlation model, which includes the power law and Lorentzian peak terms (*I*_correlation_(*q*) *=* *I*_power_(*q*) + *I*_Lorentzian_(*q*)), from eq 2 is introduced to describe the high-*q* scattering that characterizes the nanoscale structure of the SICPBs that arise due to the charge correlations:^54,55^

8 $$I_{\mathrm{correlation}}\left(q\right)=\frac{A}{q^{n}}+\frac{C}{1+{\left(\left|q-q_{0}\right|\xi \right)}^{m}}$$

where *A* is the Porod scaling factor, *n* is the Porod exponent, *C* is the Lorentzian scaling factor, *q*_0_ is the position of the Lorentzian peak, ξ is the correlation length, and *m* is the Lorentzian exponent. The first Porod term accounts for the scattering from large-scale clustering, while the second Lorentzian function describes features arising from Coulombic interactions between charged polymer segments.^24^ Disordered concentration fluctuations are present in the *r* = 0.05 and 0.10 blends as evident by the mid-*q* broad shoulders in the SAXS data shown in Figure 3b. Accordingly, we fit the SANS data to RPA model, *I*_RPA_, eq 3, to extract the thermodynamic contributions from polymer backbones, quantified by χ_SC_.

Figure 4a,b show the SANS profiles of 30 kDa dPEO/P­(LiMTFSI) at *r* = 0.05 and 0.10, respectively, across a temperature range of 70 to 130 °C, where the symbols represent the SANS data and the solid line shows the fit to eq 2. For the *r* = 0.05 blend shown in Figure 4a, the SANS intensity steadily increases with temperature indicating a strong temperature-dependence on the nanoscale structure.^16,20^ Similar changes are observed in the same *q*-region of the SAXS profiles for *r* = 0.05 as shown in Figure S9a. As *r* increases to 0.10 and ion concentration increases (Figure 4b), the intensity contribution from *I*_RPA_ diminishes in the mid-*q* region, and instead, the scattering is dominated by the high-*q* Lorentzian peak captured by *I*_Lorentzian_. Compared to the *r* = 0.05 blend (Figure 4a), temperature-dependent changes in SANS intensity are less pronounced for the *r* = 0.10 blend. For both *r* = 0.05 and 0.10 blends, the correlation peaks shift slightly toward lower *q* and broaden as the temperature increases from 70 to 130 °C, indicating a temperature-induced change in domain spacing and correlation length. Figure 4c summarizes the extracted χ_SC_ values obtained from fitting eq 3 as a function of inversion temperature. Empirically, the Flory–Huggins interaction parameter, χ, is often found to vary inversely with temperature, *T*:

9 $$\chi =A^{\prime }+\frac{B^{\prime }}{T}$$

where *A*′ and *B*′ are fitting parameters. The solid lines in Figure 4c show fits to eq 9. The value of *B*′ is negative for the *r* = 0.05 blend, indicating that the blend exhibits lower critical solution temperature (LCST) behavior. This is consistent with the increasing SANS intensities observed in Figure 4a as temperature increases.^20,56^ Previous studies of lithiated single-ion conducting block copolymers also reported LCST behavior.^45^ At low *r*, corresponding to low ion concentrations, ion-dipole interactions weaken at higher temperatures, reducing miscibility and increasing χ_SC_. As the P­(LiMTFSI) concentration increases to *r* = 0.10, the blend exhibits upper critical solution temperature (UCST) behavior, with the value of *B*′ in eq 9 becoming positive. The results in Figure 4c also show that, at most temperatures, the blends exhibit negative values of χ_SC_, consistent with favorable interactions between dPEO and P­(LiMTFSI). Only at 70 °C for the *r* = 0.10 electrolyte does χ_SC_ become positive, however even under these conditions it remains below the critical value for macrophase separation, e.g., $\chi_{crit}=\frac{1}{2}{\left(\frac{1}{\sqrt{N_{1}}}+\frac{1}{\sqrt{N_{2}}}\right)}^{2}=0.0134$ .^57^ The low values of χ_SC_ also correspond to low values of χ*N*, further supporting our claim that our SICPB system lies in the disordered regime.

**Figure 4:**
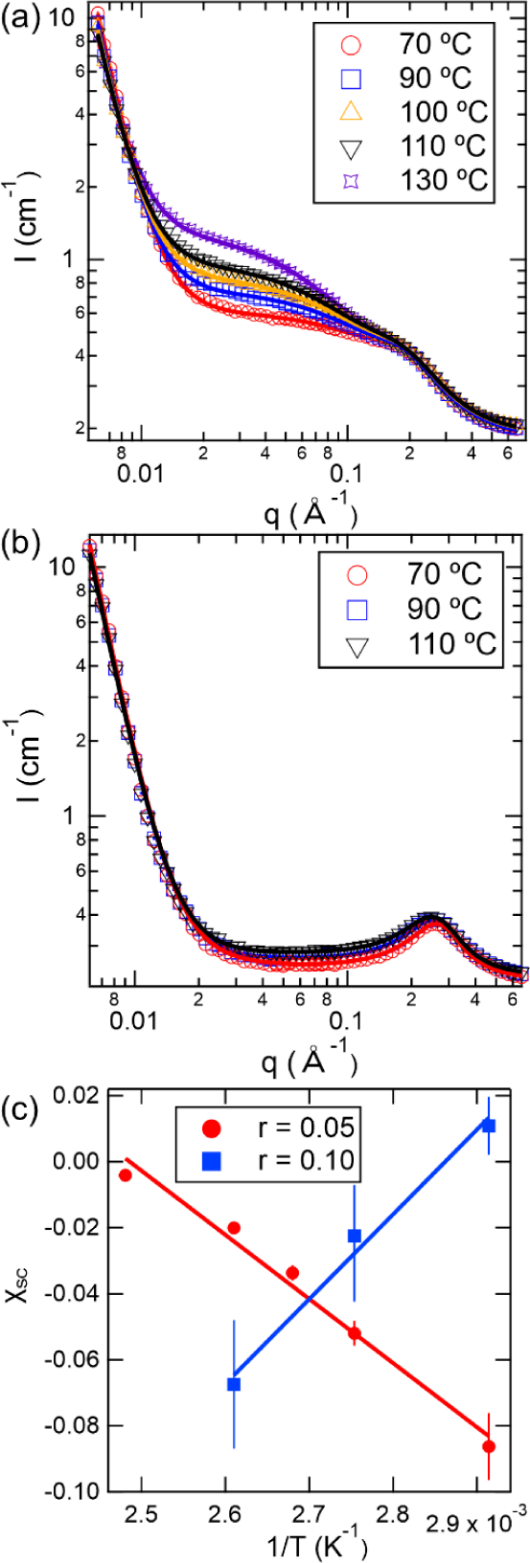
SANS profiles and corresponding fits for 30 kDa dPEO/P­(LiMTFSI) blends at various temperatures with mixing ratios of *r* = (a) 0.05 and (b) 0.10. The solid lines represent fits to eq 2. (c) Temperature dependence of χ_SC_ determined from fitting the SANS data with eq 3 for *r* = 0.05 (red) and *r* = 0.10 (blue). Error bars represent the standard deviation of the fitted value of χ_SC_. Solid lines represent linear fits to the experimental data according to eq 9.

The results shown in Figure 4c agree quantitatively with the theoretical predictions for the effective interaction parameter, χ_eff_, of charge-neutral polymer blends of Sing and co-workers,^22,30^ showing that the value of χ_SC_ and its temperature dependence vary with blend composition, *r*. In addition, their theory predicted phase separation at low volume fraction of charged polymers.^29^ Our findings support these conclusions, as the blend with the lowest composition of P­(LiMTFSI), *r* = 0.05, is located nearest to the order–disorder transition boundary. Figure 3a shows a distinct change in slope at *q* ≥ 0.3 Å^–1^ for blend with *r* ≥ 0.15, deviating from typical scaling of *q* ^–2^ found in RPA. In addition, the SAXS profiles in Figure 3b do not exhibit the broad shoulders associated with concentration fluctuations at *r* ≥ 0.15. Attempts to fit the SANS profiles for *r* = 0.15 and 0.20 using the RPA model in eq 3 did not yield reasonable values for χ_SC_, consistent with the featureless SAXS data. Therefore, contributions from *I*_RPA_ were excluded from the fitting. All fitted parameters and associated errors from the fits to eq 2 are provided in Tables S3 and S4 for 10 kDa dPEO/PLiMTFSI and 30 kDa dPEO/PLiMTFSI blends, respectively. Figure S12 shows Kratky plots, where $\frac{q^{2}S\left(q\right)}{\nu }$ is plotted against *q*, for the *r* = 0.05 blend at various temperatures to investigate the compactness of macromolecules and to calculate the statistical segment length of polymer chains.^13,19,47^ This plot shows that the SICPB system does not exhibit a plateau at the high-*q* region, reflecting a slightly swollen behavior of the SIPBE due to the electrostatic interaction between the charged side chains on P­(LiMTFSI). Similar features have been reported in charged polymer systems such as polyelectrolyte complexes^58^ and ionomer solutions^59^ due to the formation of ionic aggregates.

Figure 5 shows the results from fitting the correlation model, *I*_correlation_ (eq 8), to the SANS profiles as a function of temperature, *T*, at a variety of mixing ratios, *r*. From these fits, two characteristic length scales describing the nanoscale morphology of the charged-neutral polymer blends are extracted, as shown in Figure 5a. The interdomain spacing, $d=\frac{2\pi }{q_{0}}$ , reflects the average distance between charge-correlated domains, while the correlation length, ξ, describes the characteristic size of local charge-correlated regions. Figure 5b plots *d* as a function of temperature for various values of *r*, with solid lines representing linear fits to the data. The value of *d* increases with increasing temperature for all *r*, implying that the structural changes arise from thermal effects rather than temperature-induced phase transitions. The temperature dependence of *d* is slightly stronger for the *r* = 0.05 sample (red trace), which matches the strong temperature dependence of the SANS data shown in Figure 4a. At a given temperature, *d* decreases as *r* increases, but the magnitude of this change diminishes at higher *r*. For instance, at 70 °C, the interdomain spacing for *r* = 0.05 (red trace) is 34.9 Å, which decreases significantly to 24.0 Å at *r* = 0.10 (blue trace). Further increasing the P­(LiMTFSI) content to *r* = 0.15 (orange trace) results in a smaller decrease to 20.4 Å and the spacing remains relatively constant at 18.9 Å for *r* = 0.20 (black trace). This trend indicates that interdomain spacing is highly sensitive to the fraction of charged polymer at low P­(LiMTFSI) concentrations but becomes relatively constant at higher concentrations. A similar trend has been reported in PEO-based sulfonate ionomers,^60^ where the average interaggregate spacing also decreases as ion concentration increases, suggesting that the ionic aggregates are strongly influenced by the concentration of charged species.

**Figure 5:**
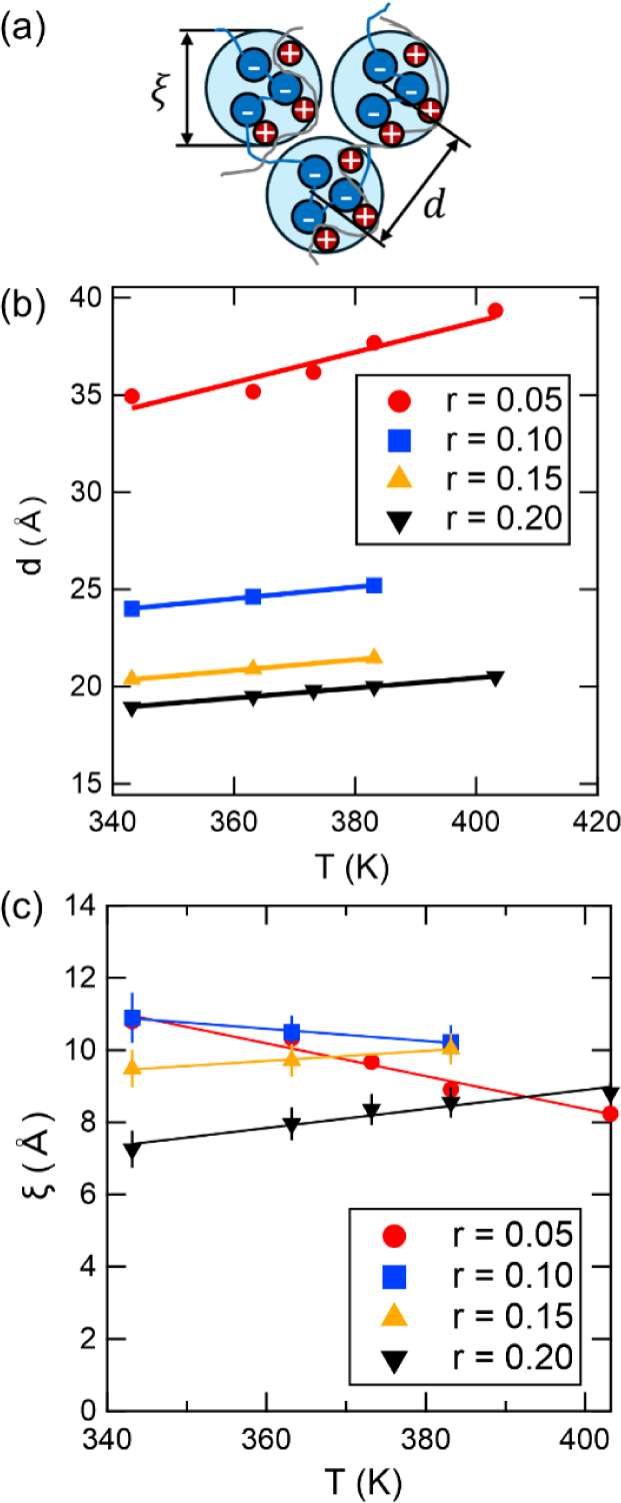
(a) Schematic illustration of charge-correlated region highlighting the interdomain spacing, *d*, and the correlation length, ξ. (b) The interdomain spacing and (c) the correlation length as a function of temperature, *T*, at various mixing ratios, *r*, of 30 kDa dPEO/P­(LiMTFSI). Solid lines represent linear fits to the experimental data. Error bars represent the standard deviation obtained from the fit to eq 8.

Figure 5c plots ξ as a function of temperature for various values of *r* considered, where solid lines illustrate the linear relationship between ξ and temperature. For *r* ≤ 0.10, ξ decreases with increasing temperature, indicating that the size of the charge correlations decreases as temperature increases. This temperature effect is more pronounced for the *r* = 0.05 sample (red trace) than for *r* = 0.10 (blue trace), similar to the trends observed for *d* . In contrast, for *r* ≥ 0.15, ξ increases with increasing temperature, indicating that charge-correlated nanostructures grow in size at elevated temperatures. In other words, as temperature increases, the size of the charge-correlated nanostructures decreases and the distance between them increases for blends with *r* ≤ 0.10. For blends with *r* ≥ 0.15, both the size and spacing of charge correlations increase with temperature. Similar trends in *r* and *T* on *d* and ξ are observed in 10 kDa dPEO/P­(LiMTFSI) blends shown in Figure S10. Compared with Figure 5, lower *M*_n,dPEO_ does not significantly affect *d* and ξ, showing that *M*_n,dPEO_ is not the primary factor influencing the thermodynamics and nanostructure of SICPBs. This finding is consistent with our DSC results, in which the fitted parameter *k*, quantifying nonideal mixing, does not change significantly between blends prepared with 10 kDa and 30 kDa dPEO. Therefore, *r* appears to have the strongest influence on blend thermodynamics.

This study provides the first experimental data set on the nanostructure of charged-neutral polymer blends and provides a useful basis for comparisons with previously developed theories. The model used to fit the SANS data includes two main contributions: the first is χ_SC_, which quantifies the thermodynamics between the polymer backbones; the second includes the structural parameters, *d* and ξ, which describe the nanoscale features induced by charge correlations. According to the theory developed by Sing and coworkers,^30^ χ_eff_ = χ – α , where α is a correction term that reflects local charge structure and is dependent on blend composition. We propose that *d* and ξ are directly related to α, and that our analysis effectively captures changes in χ_eff_ as a function of blend composition. For example, in the blend composition window where χ_SC_ was determined (*r* = 0.05 and 0.10), we observe significant differences in *d* and ξ, consistent with theoretical predictions that composition strongly influences thermodynamics and nanostructure. Unfortunately, the design of our SICPB system does not allow for independent tuning of the charge density and electrostatic interaction strength, both of which are critical parameters for understanding α and χ_eff_. Thus, we cannot precisely determine which blend properties in our charged-neutral blends drive the observed structural changes. In addition, we choose to limit our discussion to temperature-related structural changes, as temperature influences many blend properties including χ_SC_, dielectric constants,^60^ and electrostatic strengths. One parameter commonly used to quantify the electrostatic strength is the Bjerrum length, which depends on both the dielectric constant and the temperature of the system.^61^ Because these factors are inherently coupled, our experimental results do not allow us to distinguish their individual contributions to the observed structural changes. Future research will focus on designing tunable charged-neutral polymer blend systems in which the strength of charge correlations and the charge density can be precisely controlled to further investigate blend thermodynamics.

## Conclusions

We have reported the first experimental investigation of the nanostructure and phase behavior of a series of single-ion conducting polymer blends (SICPBs), dPEO/P­(LiMTFSI), using SANS and SAXS. Although all blends investigated here were macroscopically miscible, as indicated by the observation of a single *T*_g_, the structural analysis revealed nanoscale inhomogeneity arising from the interplay between charge correlations and ion-dipole interactions. At low content of charge polymer, the crystallinity of dPEO was not fully suppressed by P­(LiMTFSI), and short-range repulsion between polymers was observed to dominate, leading to disordered concentration fluctuations. As more P­(LiMTFSI) was introduced, electrostatic interactions were found to stabilize the blend morphology, enhance miscibility, and lead to a disordered phase with charge-correlated nanostructures, consistent with predictions from the SCFT-LS model previously proposed by Sing and coworkers. The temperature dependence of both the interdomain spacing and correlation length is also strongly influenced by blend composition *r*. Future work will investigate how the origin of the temperature effect influences the thermodynamics and nanostructure of SICPBs.

## Supplementary Material



## Notes

Synthetic route, NMR spectra, GPC trace, DSC, SANS, SAXS, fitting results (PDF)

## Abbreviation

*A,B*: Scaling factors of correlation model
*A*′,*B*′: Fitting parameters for temperature-dependent Flory–Huggins interaction parameter
*B*_*i*_: Neutron scattering length density of species *i*
*b*_*i*_: Neutron scattering length of species *i*
*Đ*: Dispersity
*I*(*q*): Scattering intensity
*k*: adjustable parameter of Gordon–Taylor equation
*l*_*i*_: Statistical segment length of species *i* (Å)
*M*_n,*i*_: Molecular weight of species *i*
*m*: Lorentzian exponent
*N*_*i*_: Degree of polymerization of species *i*
*n*: Porod exponent
*P*_*i*_(*q*): Form factor of species *i*
*q*: Scattering vector
*q*_0_: Lorentzian peak position
*R*_*g,i*_: Radius of gyration
*r*: Mixing ratio of P­(LiMTFSI) to dPEO (=[Li^+^]/[dEO] = [P­(LiMTFSI)]/[dPEO])
*Sii*: Structure factor of species *i*
*T*: Absolute temperature (K)
*T*_g_: Glass-transition temperature
*T*_m_: Melting temperature
*w*_*i*_: Weight fraction of species *i*
α_*i*_: Chain stretching parameter
*χ*: Flory–Huggins interaction parameter
χ_SC_: Flory–Huggins interaction parameter determined from SANS results
ξ: Correlation length of Lorentzian peak
λ: Wavelength of the X-ray/neutron
*φ*_*i*_: Volume fraction of polymer *i*
ρ*_i_*: Density of species *i* (g/cm^3^)
θ: Scattering angle
*ν*_*i*_: Molar volume of species *i*
ν_ref_: Reference volume.
