# Supplementary material for: The Influence of Charge Correlation and Ion Solvation on the Phase Behavior of Single-Ion Conducting Polymer Blend Electrolytes Using SAXS/SANS
Source: Macromolecules. 2025 Aug 11;58(16):8866–76. doi: 10.1021/acs.macromol.5c00860 (PMC12503382; doi:10.1021/acs.macromol.5c00860)
Supplement: Supplementary file 1 [file ma5c00860_si_001.pdf]

Supporting Information for:

The Influence of Charge Correlation and Ion Solvation on the Phase Behavior of Single-ion Conducting Polymer Blend Electrolytes Using SAXS/SANS

Hsin-Ju Wu[1], *Lilin He*[2], *William M. Breining*[3], *David M. Lynn*[1,3], *Whitney S. Loo*[1,*]

[1]Department of Chemical and Biological Engineering, University of Wisconsin—Madison, 1415 Engineering Drive, Madison, Wisconsin 53706, United States

[2]Neutron Scattering Division, Oak Ridge National Laboratory, Oak Ridge, Tennessee 37831-6393, United States

[3]Department of Chemistry, University of Wisconsin—Madison, 1101 University Avenue, Madison, Wisconsin 53706, United States

[*]Corresponding Author email: wloo@wisc.edu

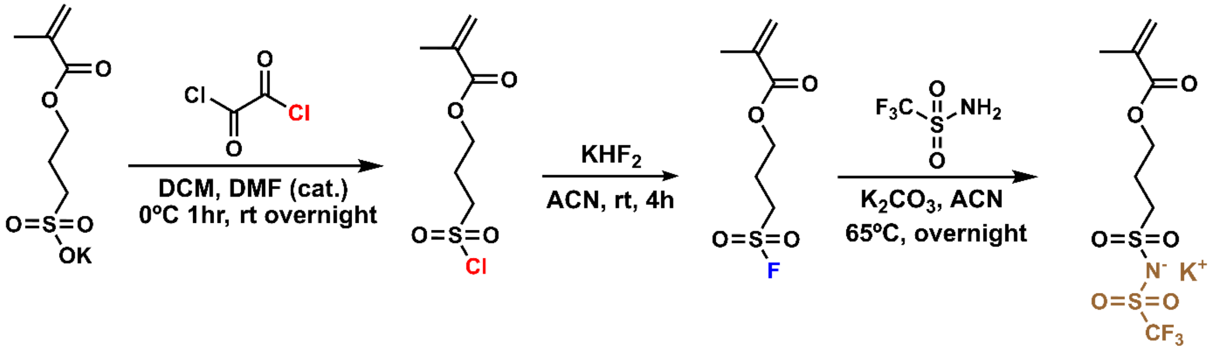

Scheme S1. Synthetic approach for sulfonylimide monomer using SuFEx click reaction.[1]

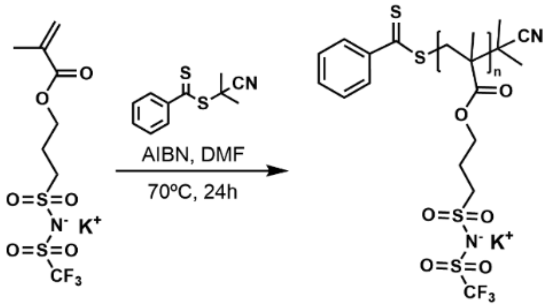

Scheme S2. RAFT polymerization of KMTFSI.

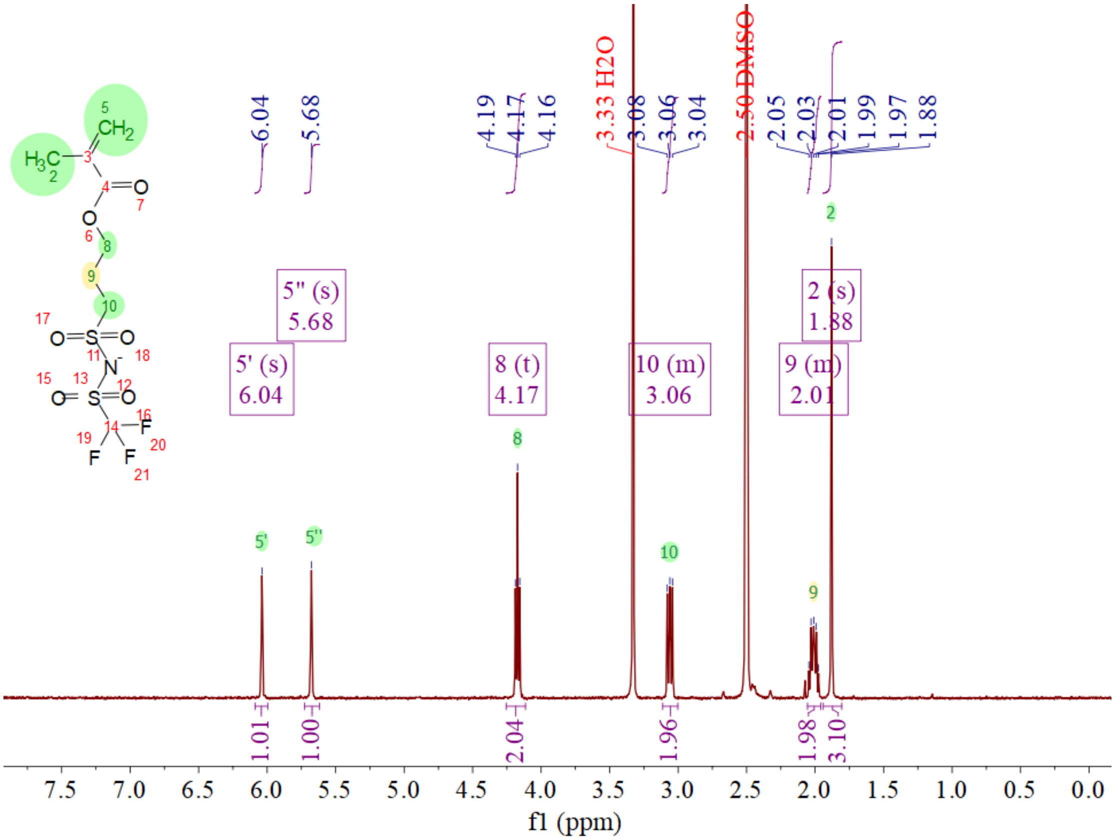

Figure S1. ^{1}H-NMR spectrum of KMTFSI monomer (400 MHz, DMSO) δ 6.04 (s, 1H), 5.68 (s, 1H), 4.17 (t, *J* = 6.4 Hz, 2H), 3.11 – 3.00 (m, 2H), 2.06 – 1.96 (m, 2H), 1.88 (s, 3H).

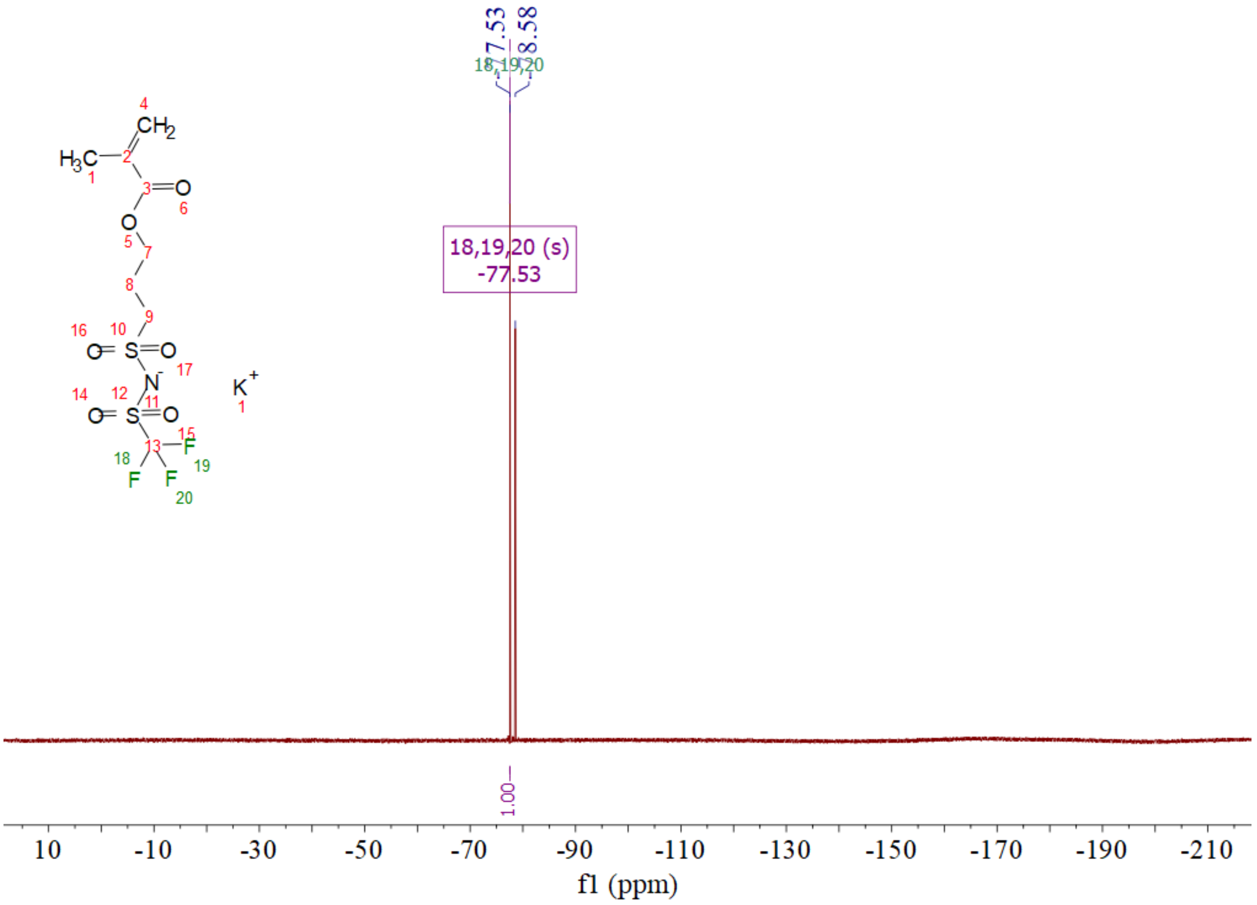

Figure S2. ^{19}F-NMR spectrum of KMTFSI monomer (377 MHz, DMSO) δ -77.53.

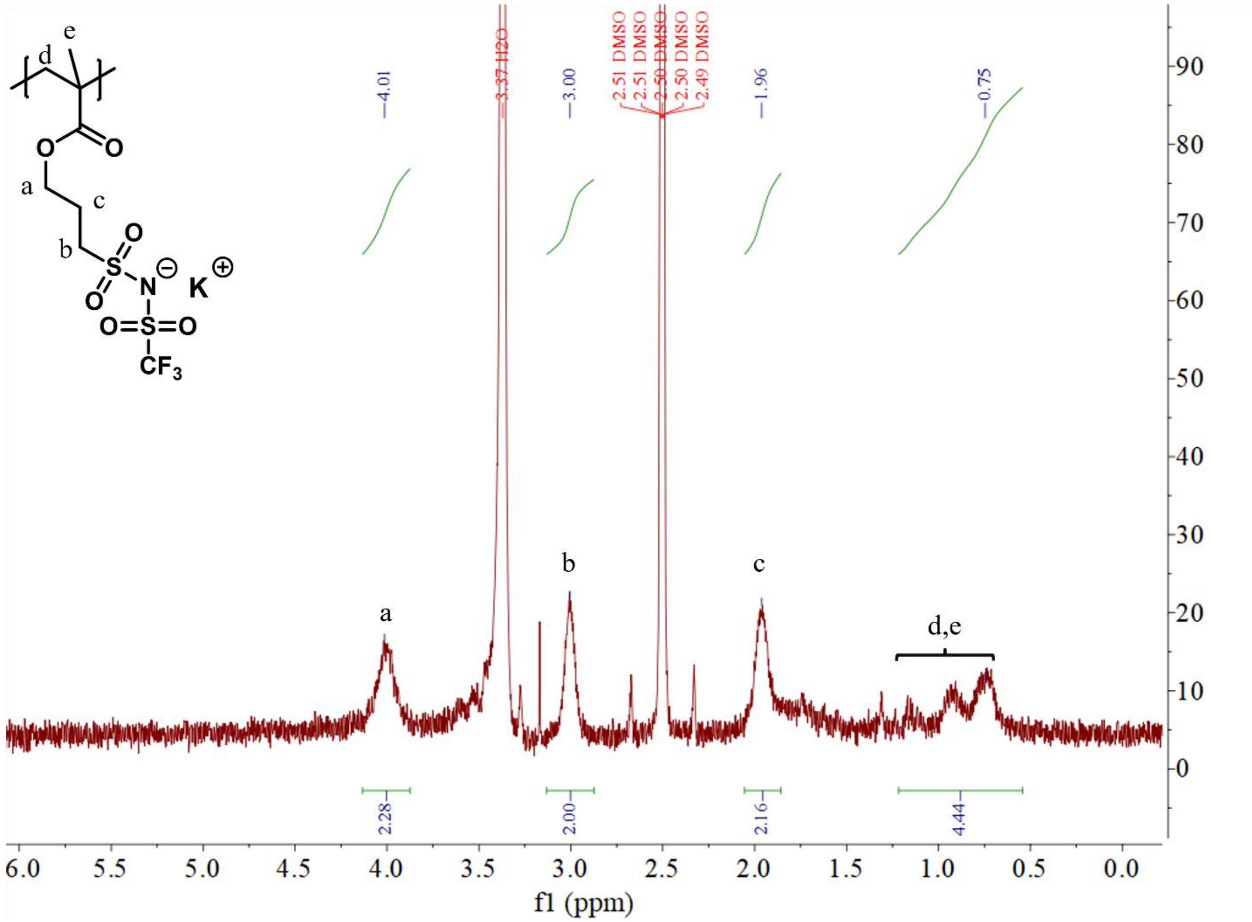

Figure S3. ^{1}H-NMR spectrum of P(LiMTFSI) (400 MHz, DMSO).

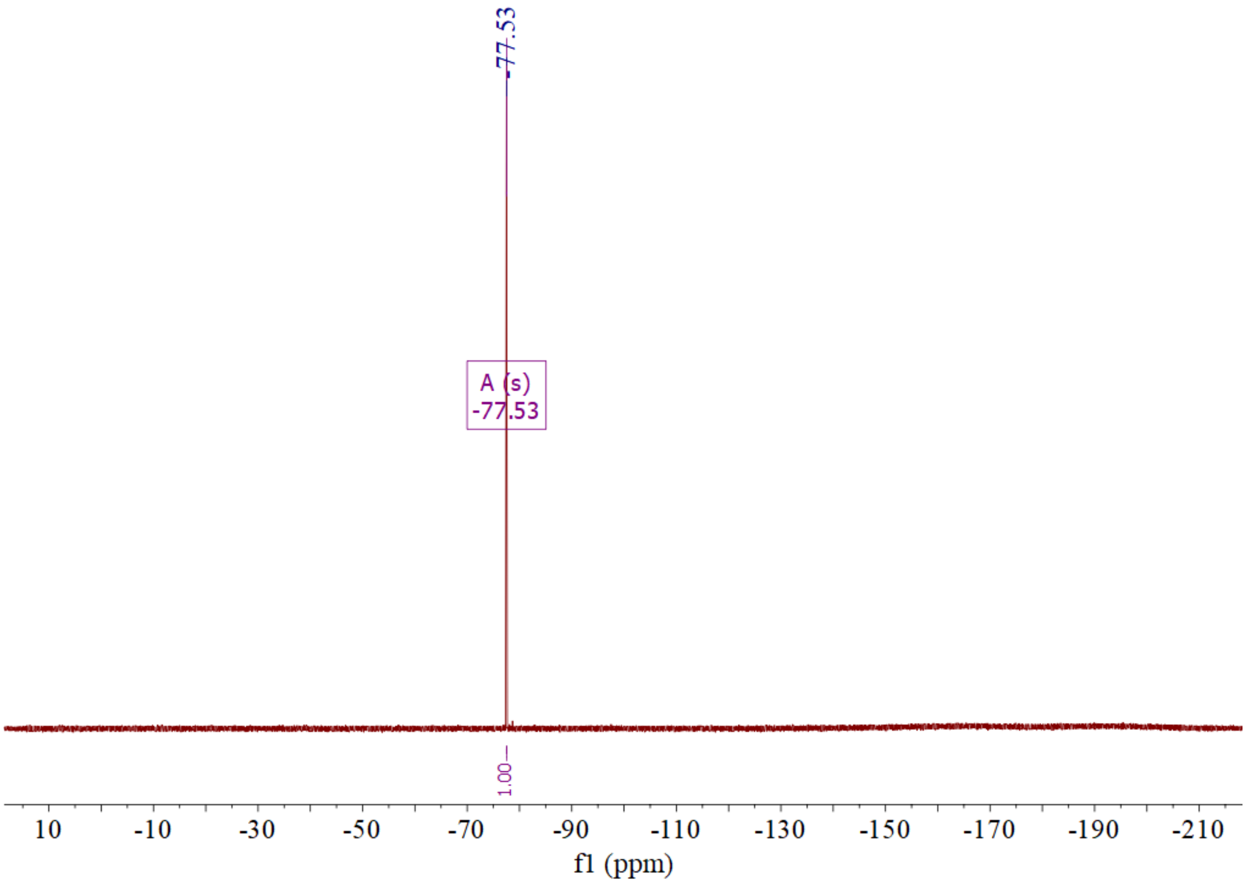

Figure S4. ^{19}F-NMR spectrum of P(LiMTFSI) (377 MHz, DMSO) δ -77.53.

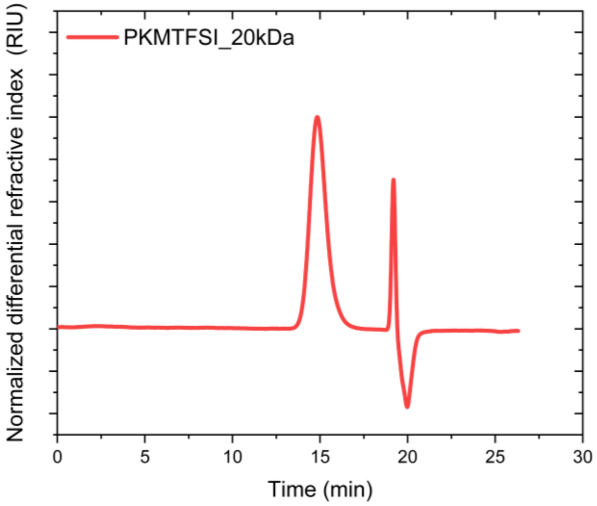

Figure S5. GPC trace of P(KMTFSI). (dn/dc = 0.0498)

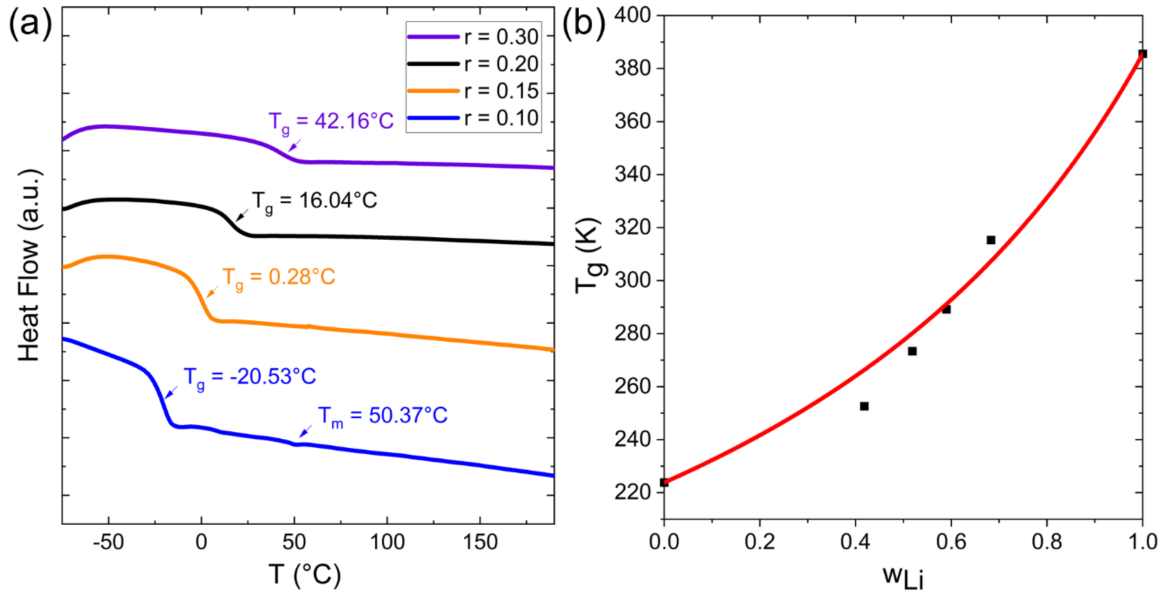

Figure S6. (a) DSC traces of 10 kDa dPEO/P(LiMTFSI) at various r. (b) The $T_{g,blend}$ of 10 kDa dPEO/P(LiMTFSI) as a function of weight fraction of P(LiMTFSI), w_{Li}. The result fitted with Gordon-Taylor equation is plotted in red, solid lines. The T_g and T_m of 10 kDa dPEO are -49.3 °C and 61.0 °C, respectively.

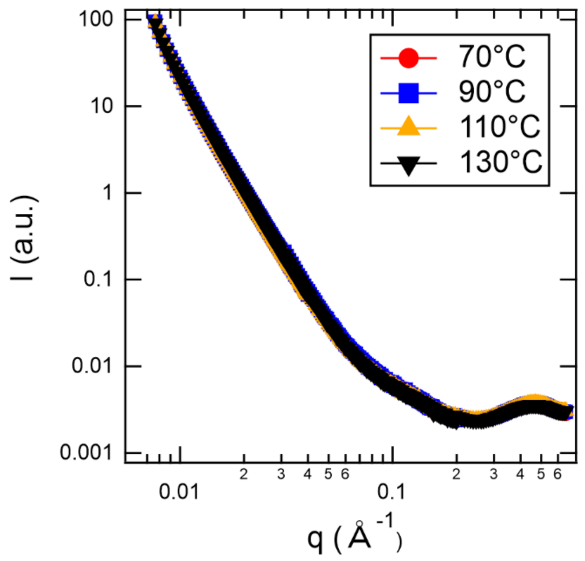

Figure S7. SAXS intensity, $I(q)$, as a function of the scattering vector, q, for pure P(LiMTFSI) polymer from 70 °C to 130 °C. Pure P(LiMTFSI) exhibits an ionic aggregation peak at 0.44 $Å^{-1}$, with its position showing minimal shift across different temperatures.

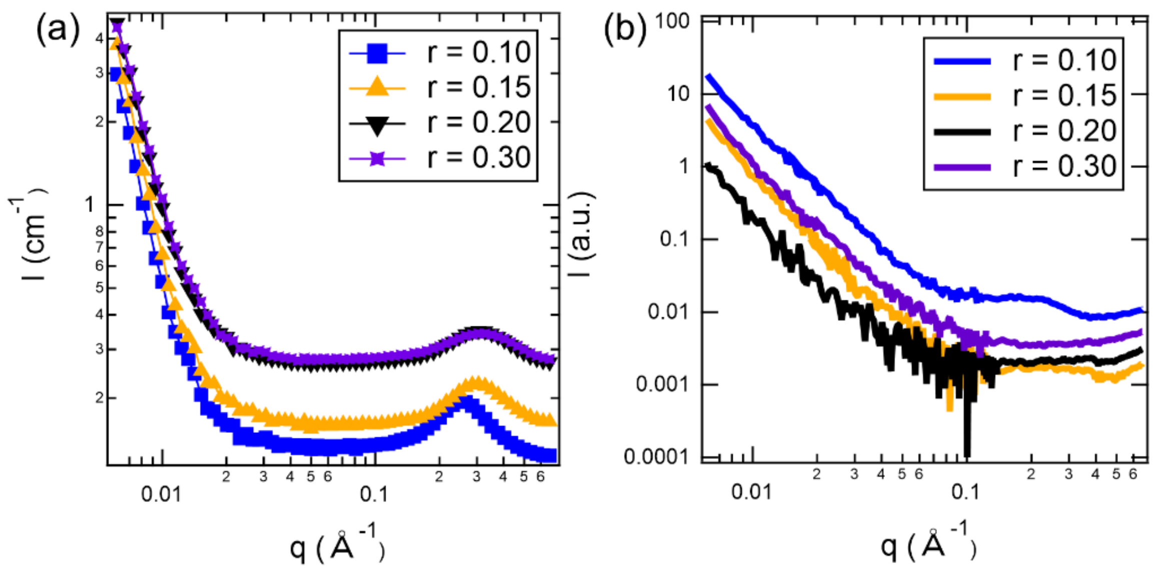

Figure S8. (a) SANS profiles for 10 kDa dPEO/P(LiMTFSI) blends at various salt concentration, r, at 70 °C. Error bars represent standard deviation. (b) SAXS intensity, $I(q)$, as a function of the scattering vector, q, for same blends at 90 °C.

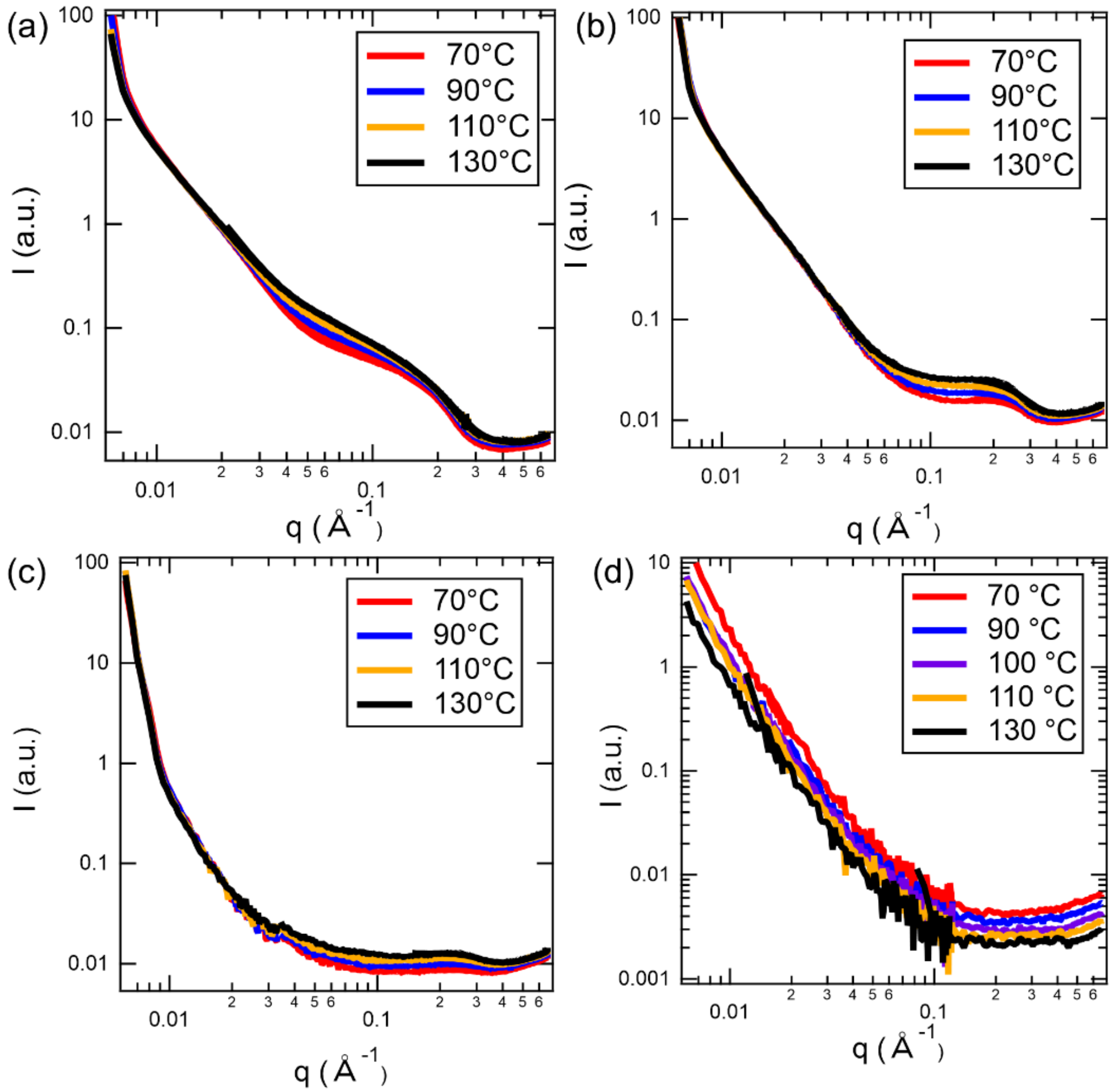

Figure S9. The combined SAXS/MAXS profiles for 30 kDa dPEO/P(LiMTFSI) blends with $r =$ (a) 0.05, (b) 0.10, (c) 0.15, and (d) 0.20 from 70 °C to 130 °C.

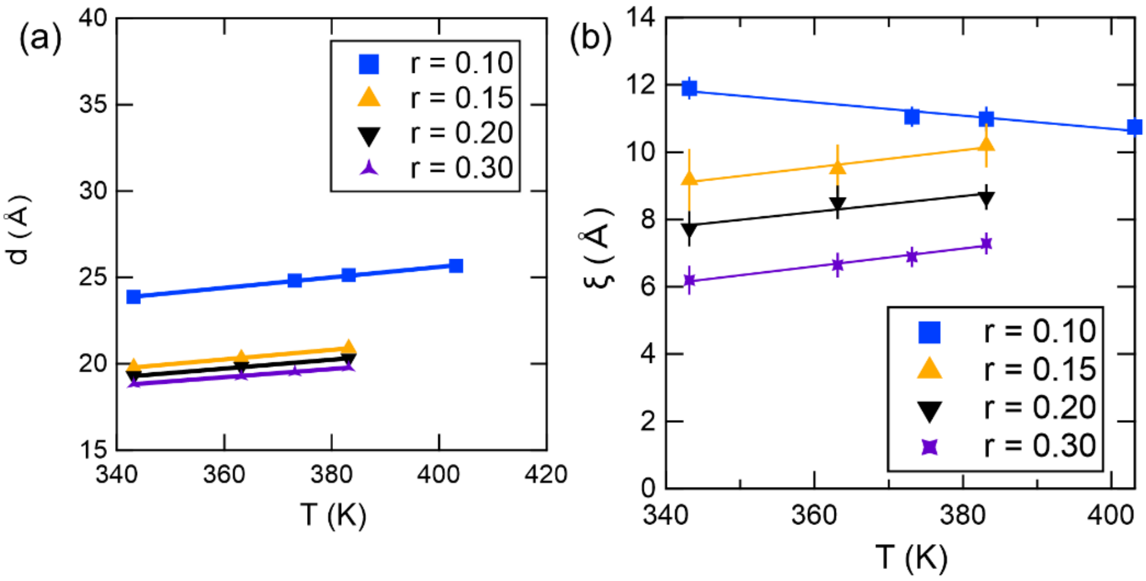

Figure S10. (a) The interdomain spacing, d, and (b) the correlation length, ξ, of charge correlation versus temperature, T, at various salt concentration, r, of 10 kDa dPEO/P(LiMTFSI). Error bars represent standard deviation.

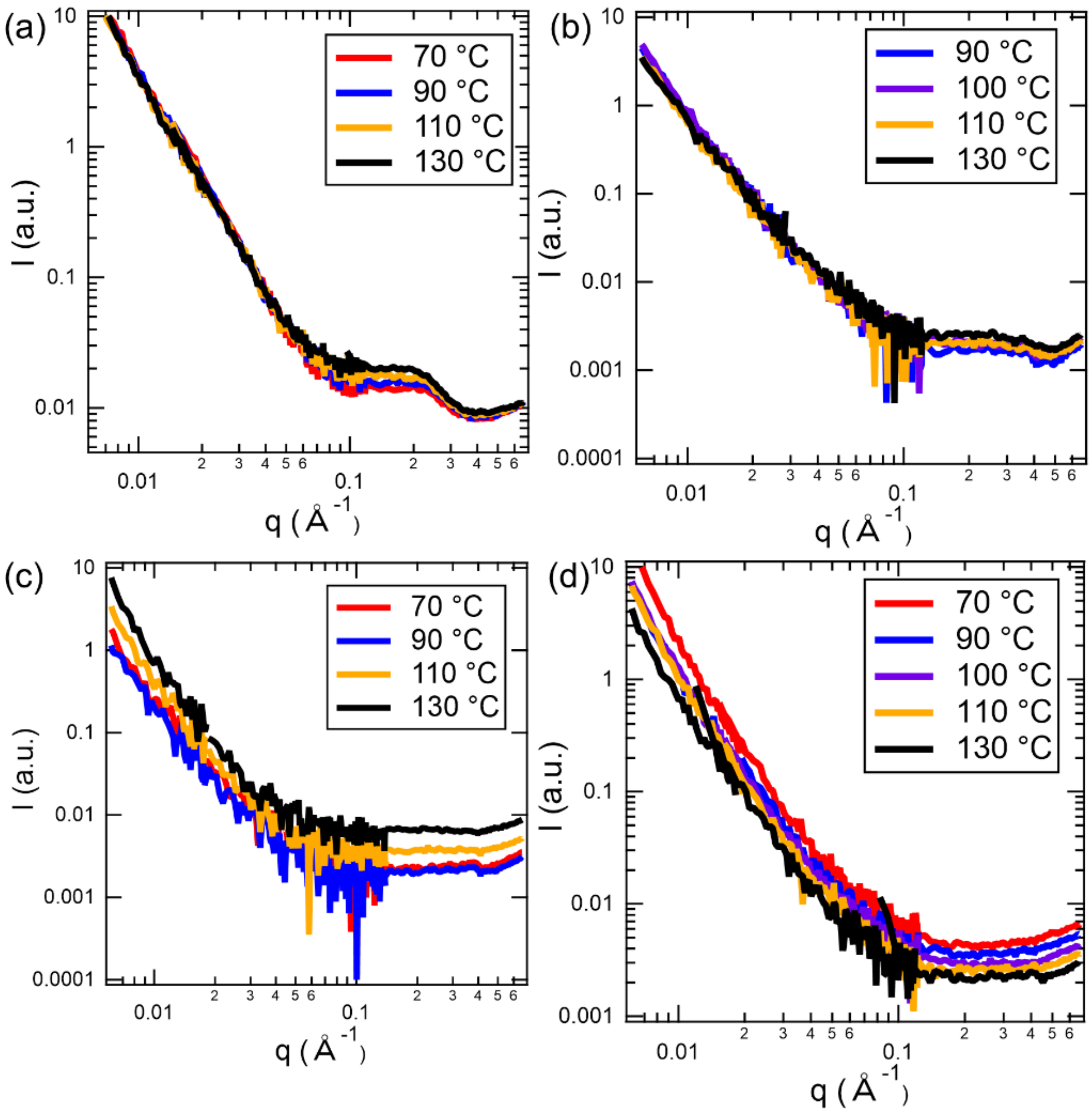

Figure S11. The combined SAXS/MAXS profiles for 10 kDa dPEO/P(LiMTFSI) blends with r = (a) 0.10, (b) 0.15, (c) 0.20, and (d) 0.30 from 70 °C to 130 °C.

To study how charge correlations affect the structural properties of SICPBs, the structure factor of SIPBE, $S(q)$, is calculated by:

$$S(q) = \frac{I_{RPA}(q)}{v_{ref}(B_{dPEO} - B_{PLiMTFSI})^2} \quad \text{(S1)}$$

The high-q regions of the SANS data can be fit with the correlation model, $I_{correlation}$, to determine how the structure of the positional charge correlations change with blend composition and temperature[2].

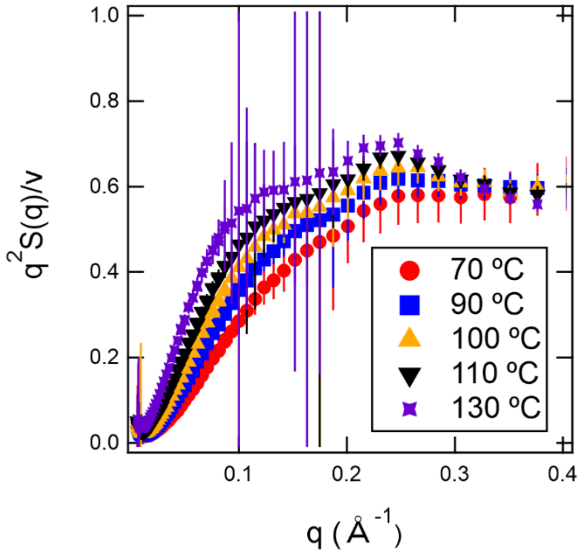

Figure S12. Kratky plot of 30 kDa dPEO/PLiMTFSI with r = 0.05 from 70 °C to 130 °C. Error bars represent propagated error from fitted parameters to I_{RPA} (**Eq (3)**) in the main text.

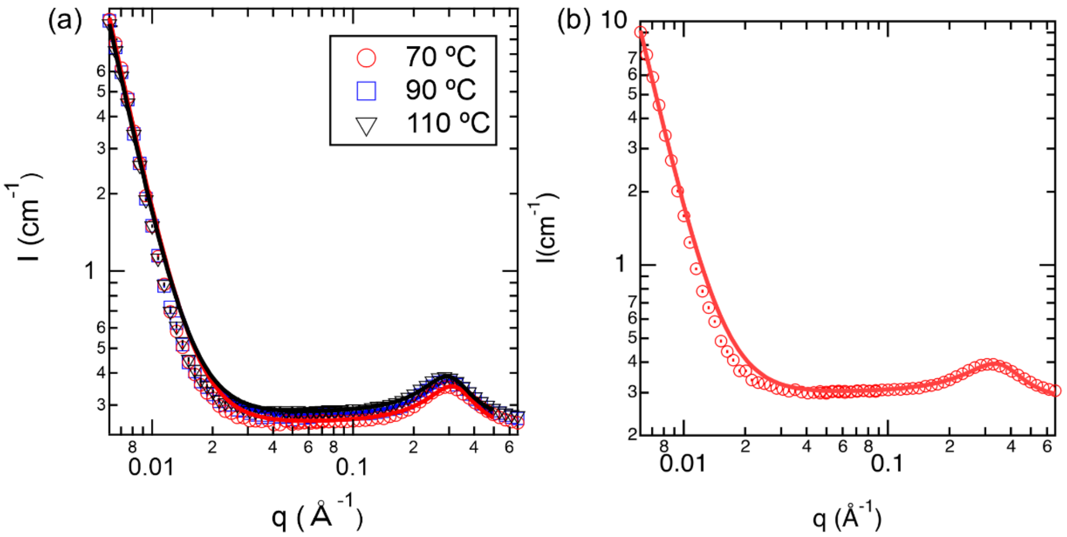

Figure S13. Representative fits to the SANS data for 30 kDa dPEO/P(LiMTFSI) blends with mixing ratios of (a) r = 0.15 and (b) r = 0.20 at 70 °C.

Table S1. Characteristics of P(KMTFSI) and dPEO. Molecular weights of P(KMTFSI) were determined by GPC. The information about dPEO was provided by Polymer Source.

Sample	M_n (kg/mol)	$Đ$	N
P(KMTFSI)	21.5	1.13	62
10 kDa dPEO	14	1.09	291
30 kDa dPEO	36	1.09	749

Table S2. The library of dPEO/P(LiMTFSI) blends. The mixing ratio, r, is calculated as the ratio of lithium ions to deuterated ethylene oxide monomers as given by $r = [Li^{+}]/[dEO] = [P(LiMTFSI)]/[dPEO]$. The weight fraction of P(LiMTFSI) in the blends is denoted as w_{Li}.

Sample name $M_{n,dPEO}/M_{n,P(LiMTFSI)}/r$	r	w_{Li}
10/20/0.10	0.10	0.418
10/20/0.15	0.15	0.519
10/20/0.20	0.20	0.590
10/20/0.30	0.30	0.683
30/20/0.05	0.05	0.264
30/20/0.10	0.10	0.418
30/20/0.15	0.15	0.519
30/20/0.20	0.20	0.590

Table S3. Fitting results of 10 kDa dPEO/PLiMTFSI blends with **Eq (2)** in the main text. Error bars represent standard deviations from the fits.

r	T(℃)	A	n	B	χ_{sc}	C	q_0	ξ	I_{inc}
0.10	70	3.62×10^{-9} ± 3.78×10^{-10}	4.02 ± 0.02	0	0	7.43×10^{-2} ± 9.53×10^{-4}	2.63×10^{-1} ± 1.03×10^{-3}	11.91 ± 0.34	1.21×10^{-1} ± 6.89×10^{-4}
	100	1.68×10^{-9} ± 3.60×10^{-10}	4.16 ± 0.04	8.83×10^{-4} ± 6.48×10^{-4}	-5.23×10^{-2} ± 5.89×10^{-2}	7.80×10^{-2} ± 9.79×10^{-4}	2.53×10^{-1} ± 1.05×10^{-3}	11.05 ± 0.30	1.23×10^{-1} ± 8.00×10^{-4}
	110	6.22×10^{-10} ± 2.04×10^{-10}	4.37 ± 0.07	1.85×10^{-3} ± 1.20×10^{-3}	-7.60×10^{-2} ± 6.57×10^{-2}	7.93×10^{-2} ± 1.27×10^{-3}	2.50×10^{-1} ± 1.30×10^{-3}	10.99 ± 0.36	1.24×10^{-1} ± 9.37×10^{-4}
	130	4.62×10^{-9} ± 8.07×10^{-10}	3.95 ± 0.03	5.99×10^{-3} ± 2.29×10^{-3}	-1.41×10^{-1} ± 5.89×10^{-2}	7.74×10^{-2} ± 1.36×10^{-3}	2.45×10^{-1} ± 1.11×10^{-3}	10.74 ± 0.31	1.21×10^{-1} ± 6.66×10^{-4}
0.15	70	1.78×10^{-8} ± 3.23×10^{-9}	3.75 ± 0.04	0	0	7.66×10^{-2} ± 3.69×10^{-3}	3.17×10^{-1} ± 3.71×10^{-3}	9.17 ± 0.92	1.50×10^{-1} ± 3.68×10^{-3}

	90	1.55×10^{-8} ± 2.79×10^{-9}	3.77 ± 0.04	0	0	8.00×10^{-2} ± 2.81×10^{-3}	3.09×10^{-1} ± 2.87×10^{-3}	9.50 ± 0.72	1.55×10^{-1} ± 2.68×10^{-3}
	110	1.32×10^{-8} ± 2.57×10^{-9}	3.80 ± 0.04	0	0	8.00×10^{-2} ± 2.49×10^{-3}	3.01×10^{-1} ± 2.31×10^{-3}	10.20 ± 0.65	1.59×10^{-1} ± 2.32×10^{-3}
0.20	70	3.75×10^{-8} ± 4.45×10^{-9}	3.65 ± 0.02	0	0	1.03×10^{-1} ± 3.41×10^{-3}	3.26×10^{-1} ± 2.63×10^{-3}	7.73 ± 0.52	2.49×10^{-1} ± 3.65×10^{-3}
	90	4.34×10^{-8} ± 6.18×10^{-9}	3.61 ± 0.03	0	0	1.03×10^{-1} ± 3.09×10^{-3}	3.17×10^{-1} ± 2.20×10^{-3}	8.50 ± 0.50	2.62×10^{-1} ± 3.21×10^{-3}
	110	2.16×10^{-8} ± 1.14×10^{-9}	3.74 ± 0.01	0	0	1.08×10^{-1} ± 2.31×10^{-3}	3.09×10^{-1} ± 1.67×10^{-3}	8.67 ± 0.38	2.65×10^{-1} ± 2.36×10^{-3}
0.30	70	1.25×10^{-7} ± 2.10×10^{-8}	3.41 ± 0.03	0	0	9.21×10^{-2} ± 3.78×10^{-3}	3.33×10^{-1} ± 2.57×10^{-3}	6.20 ± 0.43	2.52×10^{-1} ± 4.21×10^{-3}
	90	8.13×10^{-8} ± 1.06×10^{-8}	3.49 ± 0.03	0	0	9.42×10^{-2} ± 2.76×10^{-3}	3.26×10^{-1} ± 2.15×10^{-3}	6.65 ± 0.37	2.62×10^{-1} ± 3.08×10^{-3}

	100	$1.11\times10^{-7} \pm 1.60\times10^{-8}$	3.43 ± 0.03	0	0	$9.51\times10^{-2} \pm 2.18\times10^{-3}$	$3.22\times10^{-1} \pm 1.72\times10^{-3}$	6.89 ± 0.30	$2.70\times10^{-1} \pm 2.42\times10^{-3}$
	110	$8.34\times10^{-8} \pm 8.66\times10^{-9}$	3.49 ± 0.02	0	0	$9.68\times10^{-2} \pm 2.43\times10^{-3}$	$3.17\times10^{-1} \pm 1.73\times10^{-3}$	7.29 ± 0.33	$2.85\times10^{-1} \pm 2.63\times10^{-3}$
	130	$8.28\times10^{-8} \pm 1.22\times10^{-8}$	3.51 ± 0.03	0	0	$1.06\times10^{-1} \pm 1.87\times10^{-3}$	$3.11\times10^{-1} \pm 1.23\times10^{-3}$	7.50 ± 0.24	$3.08\times10^{-1} \pm 2.01\times10^{-3}$

Table S4. Fitting results of 30 kDa dPEO/PLiMTFSI blends with **Eq (2)** in the main text. Error bars represent standard deviations from the fits.

r	T(°C)	A	n	B	χ_{sc}	C	q_0	ξ	I_{inc}
0.05	70	6.07×10^{-9} ± 5.80×10^{-10}	4.16 ± 0.02	8.71×10^{-2} ± 8.96×10^{-3}	-8.63×10^{-2} ± 1.01×10^{-2}	1.29×10^{-1} ± 8.72×10^{-3}	1.80×10^{-1} ± 2.98×10^{-3}	10.80 ± 0.69	1.77×10^{-1} ± 1.62×10^{-3}
	90	4.45×10^{-9} ± 6.20×10^{-10}	4.20 ± 0.03	7.90×10^{-2} ± 4.10×10^{-3}	-5.20×10^{-2} ± 3.74×10^{-3}	1.26×10^{-1} ± 5.26×10^{-3}	1.79×10^{-1} ± 2.47×10^{-3}	10.33 ± 0.47	1.80×10^{-1} ± 1.28×10^{-3}
	100	6.16×10^{-9} ± 1.11×10^{-9}	4.12 ± 0.04	7.02×10^{-2} ± 3.08×10^{-3}	-3.37×10^{-2} ± 2.35×10^{-3}	1.36×10^{-1} ± 5.17×10^{-3}	1.74×10^{-1} ± 2.92×10^{-3}	9.68 ± 0.47	1.85×10^{-1} ± 1.76×10^{-3}
	110	6.71×10^{-9} ± 3.95×10^{-10}	4.10 ± 0.01	6.24×10^{-2} ± 2.17×10^{-3}	-2.01×10^{-2} ± 1.42×10^{-3}	1.45×10^{-1} ± 4.60×10^{-3}	1.67×10^{-1} ± 2.89×10^{-3}	8.91 ± 0.37	1.86×10^{-1} ± 1.75×10^{-3}
	130	8.11×10^{-9} ± 1.20×10^{-9}	4.04 ± 0.03	5.62×10^{-2} ± 1.66×10^{-3}	-4.08×10^{-3} ± 8.12×10^{-4}	1.47×10^{-1} ± 4.97×10^{-3}	1.60×10^{-1} ± 3.84×10^{-3}	8.24 ± 0.43	1.86×10^{-1} ± 2.45×10^{-3}

0.10	70	$6.11\times10^{-9} \pm 9.13\times10^{-10}$	4.20 ± 0.03	$3.78\times10^{-4} \pm 1.72\times10^{-4}$	$1.08\times10^{-2} \pm 8.76\times10^{-3}$	$1.47\times10^{-1} \pm 1.76\times10^{-3}$	$2.62\times10^{-1} \pm 8.98\times10^{-4}$	10.90 ± 0.25	$2.21\times10^{-1} \pm 1.55\times10^{-3}$
	90	$7.08\times10^{-9} \pm 9.79\times10^{-10}$	4.16 ± 0.03	$1.52\times10^{-3} \pm 5.49\times10^{-4}$	$-2.24\times10^{-2} \pm 1.99\times10^{-2}$	$1.56\times10^{-1} \pm 1.35\times10^{-3}$	$2.55\times10^{-1} \pm 7.40\times10^{-4}$	10.49 ± 0.19	$2.26\times10^{-1} \pm 1.19\times10^{-3}$
	110	$8.22\times10^{-9} \pm 1.36\times10^{-9}$	4.13 ± 0.03	$4.81\times10^{-3} \pm 1.01\times10^{-3}$	$-6.75\times10^{-2} \pm 1.95\times10^{-2}$	$1.61\times10^{-1} \pm 1.14\times10^{-3}$	$2.49\times10^{-1} \pm 6.02\times10^{-4}$	10.22 ± 0.15	$2.27\times10^{-1} \pm 8.53\times10^{-4}$
0.15	70	$4.78\times10^{-8} \pm 1.18\times10^{-8}$	3.75 ± 0.05	0	0	$1.14\times10^{-1} \pm 4.26\times10^{-3}$	$3.08\times10^{-1} \pm 2.85\times10^{-3}$	9.48 ± 0.47	$2.41\times10^{-1} \pm 4.16\times10^{-3}$
	90	$4.68\times10^{-8} \pm 1.02\times10^{-8}$	3.75 ± 0.04	0	0	$1.21\times10^{-1} \pm 2.97\times10^{-3}$	$3.01\times10^{-1} \pm 1.98\times10^{-3}$	9.71 ± 0.52	$2.53\times10^{-1} \pm 2.81\times10^{-3}$
	110	$4.59\times10^{-8} \pm 9.49\times10^{-9}$	3.75 ± 0.04	0	0	$1.25\times10^{-1} \pm 2.43\times10^{-3}$	$2.93\times10^{-1} \pm 1.42\times10^{-3}$	10.03 ± 0.39	$2.63\times10^{-1} \pm 2.28\times10^{-3}$
0.20	70	$6.42\times10^{-8} \pm$	$3.68 \pm$	0	0	$1.15\times10^{-1} \pm$	$3.32\times10^{-1} \pm$	$7.26 \pm$	$2.79\times10^{-1} \pm$

		1.25×10^{-8}	0.04			4.12×10^{-3}	2.78×10^{-3}	0.52	4.51×10^{-3}
	90	2.99×10^{-8} ± 5.58×10^{-9}	3.83 ± 0.04	0	0	1.18×10^{-1} ± 3.27×10^{-3}	3.22×10^{-1} ± 2.23×10^{-3}	7.95 ± 0.46	2.95×10^{-1} ± 3.47×10^{-3}
	100	6.53×10^{-8} ± 1.65×10^{-8}	3.67 ± 0.05	0	0	1.19×10^{-1} ± 3.00×10^{-3}	3.17×10^{-1} ± 1.96×10^{-3}	8.36 ± 0.43	3.06×10^{-1} ± 3.12×10^{-3}
	110	8.46×10^{-8} ± 1.95×10^{-8}	3.64 ± 0.05	0	0	1.30×10^{-1} ± 3.29×10^{-3}	3.14×10^{-1} ± 1.86×10^{-3}	8.55 ± 0.43	3.32×10^{-1} ± 3.39×10^{-3}
	130	3.16×10^{-8} ± 5.36×10^{-9}	3.82 ± 0.03	0	0	1.32×10^{-1} ± 2.12×10^{-3}	3.06×10^{-1} ± 1.26×10^{-3}	8.82 ± 0.29	3.27×10^{-1} ± 2.14×10^{-3}

REFERENCES

(1) Lee, O. A.; McBride, M. K.; Ticknor, M.; Sharpes, J.; Hayward, R. C. Pendent Sulfonylimide Ionic Liquid Monomers and Ionoelastomers via SuFEx Click Chemistry. *Chem. Mater.* **2023**, *35* (23), 10030–10040. https://doi.org/10.1021/acs.chemmater.3c02038.

(2) Fang, Y. N.; Rumyantsev, A. M.; Neitzel, A. E.; Liang, H.; Heller, W. T.; Nealey, P. F.; Tirrell, M. V.; de Pablo, J. J. Scattering Evidence of Positional Charge Correlations in Polyelectrolyte Complexes. *Proc. Natl. Acad. Sci.* **2023**, *120* (32), e2302151120. https://doi.org/10.1073/pnas.2302151120.
